# On the mechanism of *Candida tropicalis* biofilm reduction by the combined action of naturally-occurring anthraquinones and blue light

**DOI:** 10.1371/journal.pone.0181517

**Published:** 2017-07-19

**Authors:** Juliana Marioni, Roger Bresolí-Obach, Montserrat Agut, Laura R. Comini, José L. Cabrera, María G. Paraje, Santi Nonell, Susana C. Núñez Montoya

**Affiliations:** 1 IMBIV, CONICET and Departamento de Ciencias Farmacéuticas, Facultad de Ciencias Químicas, Universidad Nacional Córdoba, Córdoba, Argentina; 2 Institut Químic de Sarrià, Universitat Ramon Llull, Barcelona, Spain; 3 IMBIV, CONICET and Cátedra de Microbiología, Facultad de Ciencias Exactas Físicas y Naturales, Universidad Nacional de Córdoba, Córdoba, Argentina; Massachusetts General Hospital, UNITED STATES

## Abstract

The photoprocesses involved in the photo-induced *Candida tropicalis* biofilm reduction by two natural anthraquinones (AQs), rubiadin (**1**) and rubiadin-1-methyl ether (**2**), were examined. Production of singlet oxygen (^1^O_2_) and of superoxide radical anion (O_2_^•−^) was studied. Although it was not possible to detect the triplet state absorption of any AQs in biofilms, observation of ^1^O_2_ phosphorescence incubated with deuterated Phosphate Buffer Solution, indicated that this species is actually formed in biofilms. **2** was accumulated in the biofilm to a greater extent than **1** and produced measurable amounts of O_2_^•−^ after 3h incubation in biofilms. The effect of reactive oxygen species scavengers on the photo-induced biofilm reduction showed that Tiron (a specific O_2_^•−^ scavenger) is most effective than sodium azide (a specific ^1^O_2_ quencher). This suggests that O_2_^•−^ formed by electron transfer quenching of the AQs excited states, is the main photosensitizing mechanism involved in the photo-induced antibiofilm activity, whereas ^1^O_2_ participation seems of lesser importance.

## Introduction

Antimicrobial photodynamic therapy is an emerging approach for treating infections in which activation of a photosensitising substance by light in the presence of oxygen increases the levels of reactive oxygen species (ROS) and induces oxidative stress in the pathogenic microorganisms [1,2]. Thus, the scrutiny of new photosensitisers (PSs), particularly of natural origin, is a research area currently under intensive development [2,3]. Our research team is interested in investigating the potential use in antimicrobial photodynamic therapy of a group of natural 9,10-anthraquinone aglycones (AQs) with photosensitizing properties.

*Candida* species are the yeasts most commonly associated with hospital-acquired infections, which cause both superficial and systemic diseases [1,4,5]. The well-known resistance of *Candida* spp. to conventional antifungal therapies is a complex multifactorial phenomenon, caused in part by the development of biofilms [1,4–7]. Although *C*. *albicans* is the main etiologic agent of hospital-acquired infections, *C*. *tropicalis* has been noted as one of the most frequently isolated pathogenic yeasts in nosocomial candidiasis in recent years, especially in Latin America [8,9]. *C*. *tropicalis* biofilms is usually related with patients with neutropenia and malignancy, and even associated with higher mortality than other *Candida* no *albicans* spp. [4,5,7–9].

**Fig 1:**
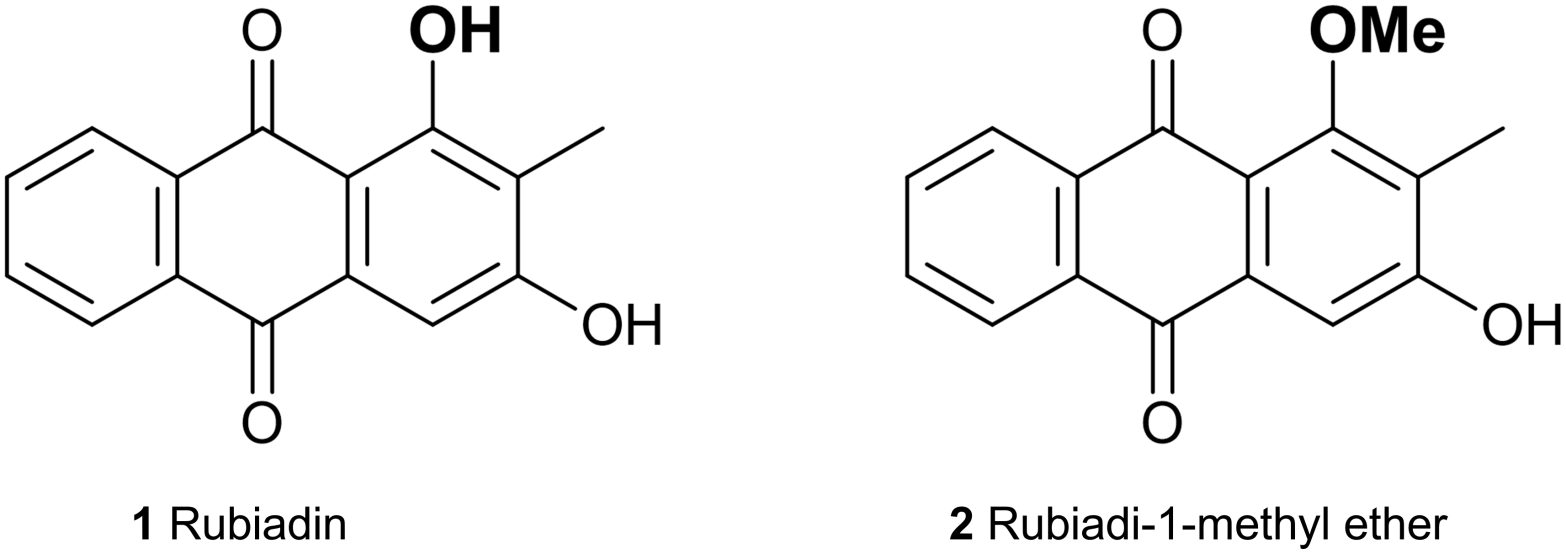
Chemical structure of rubiadin (1) and rubiadin-1-methyl ether (2).

Rubiadin (**1**) and rubiadin-1-methyl ether (**2**) (Fig 1) are two AQs isolated from the phototoxic plant *Heterophyllaea pustulata* Hook f. (*Rubiaceae*) [10,11]. This plant species grows in the Andean region of northwestern Argentina and Bolivia, where is popularly known as “cegadera”, “ciegadera” o “saruera” [12]. Photoactivation of a *H*. *pustulata* extract containing both AQs produced a reduction of 39.3 ± 3.5% in the growth of *C*. *tropicalis* biofilms. The concentration necessary to inhibit the biofilm (Biofilm Inhibitory Concentration) [13] was 0.2 mg/mL. Meanwhile, both purified AQs, **1** and **2**, showed greater antibiofilm effect than the photoactive extract under irradiation, which was mediated by a strong redox imbalance. Rubiadin was more active than its methylated derivative, since lower concentration was required to achieve similar values of reduction (1.96 μg/mL for **1** and 15.6 μg/mL for **2**, Table 1 and 2 in S1 File) [14].

We have previously established that the two AQs possess interesting photophysical and photosensitising properties [15, 16]. Both compounds absorb in the UV and blue regions of the spectrum (i.e. maximum absorbance at 411 and 360 nm, respectively) and are poorly fluorescent (*Φ*_F_ ≈ 10^−2^) in chloroform (CHCl_3_).

In this work, we set out to identify the reactive intermediates involved in the photo-induced *C*. *tropicalis* biofilm reduction by two natural AQs: rubiadin and rubiadin-1-methyl ether.

## Materials and methods

### Chemicals

The following chemicals were used as received: Crystal Violet (CV, Anedra Tigre, Argentina); Fetal Bovine Serum (Greiner Bio-One, Frickenhausen, Germany); Sabouraud Dextrose Broth (SDB, Difco, Detroit, MI); Sabouraud dextrose agar (Difco, Detroit, MI), Phosphate Buffer Solution (PBS); dimethyl sulfoxide (DMSO, Merck Darmstadt, Germany); Calcofluor-White (Sigma-Aldrich Co, St Louis, MO, USA); MeOH (HPLC grade, Merck, Germany); Nitro Blue Tetrazolium (NBT, Sigma-Aldrich Co, St Louis, MO, USA); Methionine (Sigma-Aldrich); Riboflavin (Sigma-Aldrich Co, St Louis, MO, USA), Tiron (Sigma-Aldrich); Sodium azide (Sigma-Aldrich).

Deuterium oxide (99.9%) was purchased from Solvents Documentation Synthesis (Peypin, France). Deuterated PBS (D-PBS) was prepared by dissolving PBS powder in deuterium oxide.

**Fig 2:**
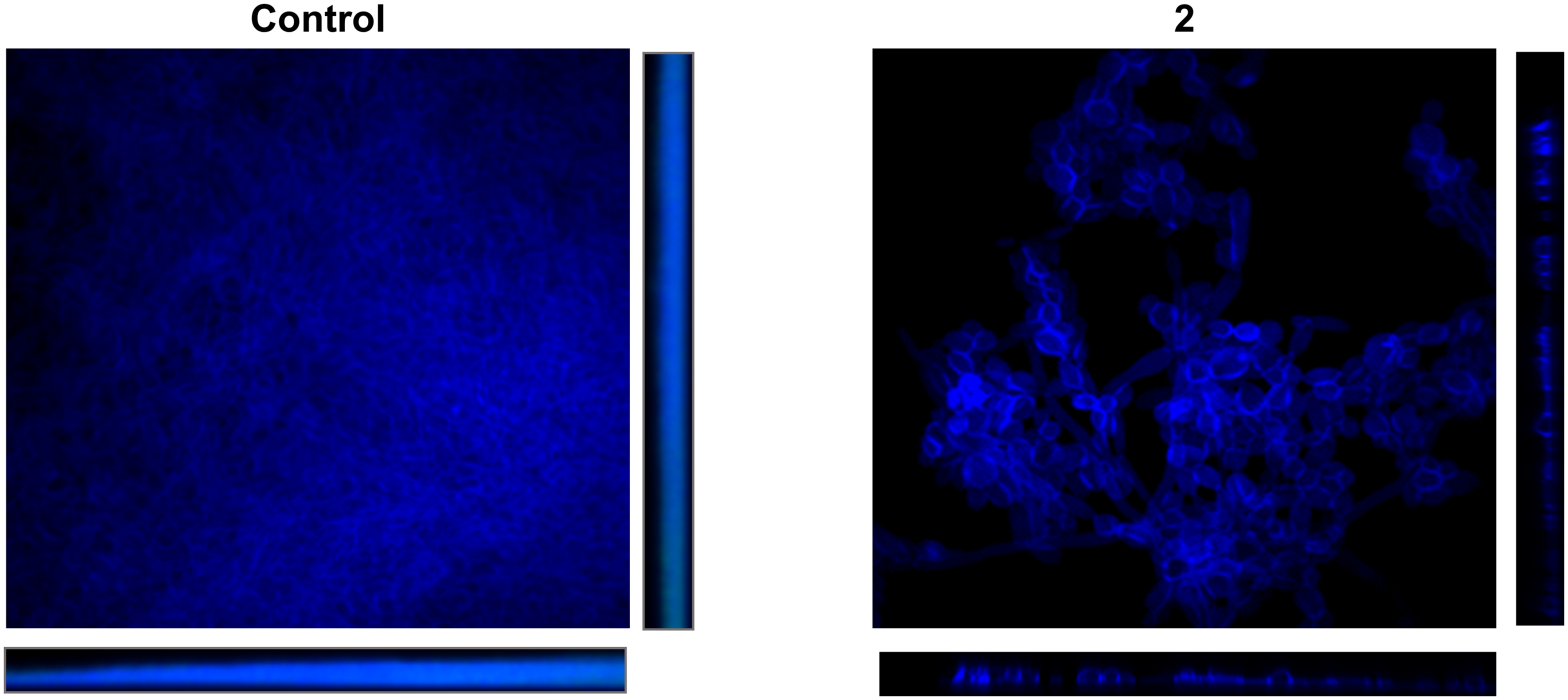
CSLM images showing the effect of rubiadin 1-methyl ether and blue light on *C*. *tropicalis* NCPF 3111 biofilms. **(A) Positive control, (B) rubiadin 1-methyl ether (56 μM)**. Blue channel shows Calcofluor white dying sessile cells walls. Magnification 60X and scale bar is 10 μm.

**1** and **2** were purified from benzene extracts of *H*. *pustulata* using a methodology described previously [10,11]. They were unequivocally identified by their spectroscopic/spectrometric data (^1^H NMR, ^13^C NMR, IR, UV–Vis, MS) [10]. The purity was 93.6% ± 0.1% for **1** and 93.8 ± 0.1% for **2**, as established by HPLC analysis [13] (Fig 2 in S1 File).

### Yeast strain conditions

Stock solution of *C*. *tropicalis* NCPF 3111 (National Collection of Pathogenic Fungi, Bristol, UK, strain N° 2), suspended in SDB with 10% glycerol (cryoprotectant), was kept at −80°C. In order to ensure purity and viability of yeasts before its use, they were plated in Sabouraud dextrose agar and then were incubated overnight in Falcon tubes at 37°C with SDB [14,17].

### Photosensitiser solutions

Each stock AQs solution was prepared in SDB with 1% DMSO. Then, a dilution with the culture medium was performed to obtain a final concentration of 56 μM for each AQ, and these solutions were stored in the dark at 4°C. All solutions were prepared and handled under light-restricted conditions.

DMSO was used to solubilize drugs in aqueous solutions, as this is a common *in vitro* practice to deliver hydrophobic drugs to cells. DMSO is a good scavenger for hydroxyl radicals, but has no effect on ^1^O_2_ at the concentration used in our studies [18]. In addition, 1% DMSO has no effect either on the rate of O_2_^•−^ production by neutrophils [19].

### Light sources

An actinic Phillips 20 W lamp (380–480 nm, emission maximum at 420 nm, 0.65 mW cm^-2^) was used in photo-induced antibiofilm studies, which was placed 20 cm above the samples inside a black box [13,14].

### *In vitro* photo-induced biofilm reduction assays

Biofilm formation was achieved following the previously published procedure [13,14,20], which is an adaptation from the method of O’Toole & Kolter (1998) [21] using flat-bottomed 96-well microplates (Greiner Bio-One, Germany). In this assay, we used the *C*. *tropicalis* NCPF 3111 strain previously classified as a strong biofilm producer [13,22]. A strain suspension (1 × 10^7^ cells/mL) in SDB was inoculated in pre-treated microplates with 50% (v/v) Fetal Bovine Serum [19], and then were incubated 90 min at 37°C. After removing the non-adhered cells and add fresh culture medium, the incubation of microplates was resumed at the same temperature during 48 h without stirring to obtain a dense biofilm [13]. Formation of the biofilm was demonstrated by confocal laser scanning microscopy (CLSM, Fig 2) [13,20,23]. Each AQs solution (56 μM) was added in triplicate onto the dense biofilm, pre-washed with sterile PBS. Control wells, containing only SDB and 1% DMSO at pH = 6.5, were included in triplicate. Two replicates of the microplates were prepared, whereas one was kept in the dark, the other was irradiated for 15 min [13,14]. Subsequently, both microplates were incubated 48 h at 37°C. After incubation, supernatant was separated to assess the production of O_2_^•−^ and SOD activity [13,14]. The total biomass of the biofilm formed was quantified by staining with a CV solution (1% w/v).To this end, the excess dye was first removed by washing with PBS and CV was extracted from the biomass with an ethanol/acetone (70:30) solution. Optical density (OD) was measured at 595 nm by using a microplate reader (Tecan Sunrise Model, TECAN, AUS). The average OD of all tested wells (n = 3) was obtained by subtracting the average OD from control wells (ODc). The biomass of the formed biofilm was expressed in biofilm biomass units (BBU), defined as 0.1 OD_595_ corresponding to 1 BBU [13,20].

### AQ accumulation assay in biofilms

Dense biofilms formed as described above in 24-well microplates, were exposed to 56 μM of **1** and **2** in SBD, respectively in duplicate, during 0.5, 1, 3 and 6 h. Control samples (in duplicate) were processed without AQ under the same working conditions. After incubation at 37°C, biofilms of each microwell (treated and controls) were collected separately by scraping. Each pellet obtained by centrifugation at 5000 rpm for 25 min, was suspended in CHCl_3_ (1 mL) and was exposed alternatively to cold and heat, using liquid air and a thermostatic water-bath at 70 °C. This procedure was repeated three times in order to break down the biofilm structures. CHCl_3_ was then used to disrupt the phospholipid bilayer of the cell membranes and extract the AQs from the biofilms [24], since their high logP values (2.26 for **1** and 2.83 for **2**) [25] indicate their preferential solubility in nonpolar solvents and accumulation in lipophilic compartments. The supernatant was evaporated to dryness and was dissolved in methanol for subsequent analysis by HPLC, following the procedure described before [13]. Once the scraping was finished, the microplate was stained with CV to verify that all biofilms were removed.

### Spectroscopic measurements

Ground-state absorption spectra were recorded using a Varian Cary 6000i spectrophotometer (Varian Inc., Palo Alto, CA). Fluorescence emission spectra were recorded in a Spex Fluoromax-4 spectrofluorometer (Horiba Jobin-Yvon, Edison, NJ). Time resolved fluorescence decays were recorded with a time-correlated single photon counting system (Fluotime 200, PicoQuant GmbH, Berlin, Germany) equipped with a red-sensitive photomultiplier. The fluorescence was excited at 375 nm by means of a pulsed laser diode working at 10 MHz repetition rate, and was observed at 420 nm keeping the counting frequency below 1%. Fluorescence decays were analysed using the PicoQuant FluoFit 4.5 data analysis software (PicoQuant, Germany) [26].

^1^O_2_ phosphorescence at 1275 nm was detected by means of a customized PicoQuant Fluotime 200 system using a diode-pumped pulsed Nd:YAG laser (FTSS355-Q, Crystal Laser, Berlin, Germany) working at 10 kHz repetition rate at 355 nm (6 mW, 0.6 μJ per pulse) for excitation. A 1064 nm rugate notch and a Schott KG5 filter (Edmund Optics, UK) were placed at the exit port of the laser to remove any residual component of its fundamental emission in the near-IR region. The emitted luminescence was filtered by using a cold mirror (CVI Melles Griot, USA) and an interference filter at 1273 ± 86 nm (Interferenzoptik Elektronik GmbH, Germany) and focused onto a near-IR sensitive photomultiplier tube assembly (H9170-45, Hamamatsu Photonics Hamamatsu City, Japan). Photon counting was achieved with a NanoHarp 250 multichannel scaler (PicoQuant, Germany). The time-resolved emission signals were likewise analyzed using the FluoFit 4.5 software to extract lifetime values [26]. ^1^O_2_ production quantum yields (Φ_Δ_) were determined by comparing the intensity of the signals to that of a standard measured under matched conditions [26]. Phenalenone, for which Φ_Δ_ = 0.95–1.0 in many solvents was used as standard [27,28].

Transient absorption experiments were carried out using a home-built nanosecond laser flash photolysis apparatus described elsewhere [27,28]. The excitation wavelength was 355 nm and the transient absorption was recorded at the appropriate wavelengths for monitoring the triplet state (^3^AQ, 675 nm), the ketyl radical (AQ^•^, 450 nm) or the radical anion (AQ^•−^, 550 nm) of the rubiadins [29].

Dense biofilms were developed on a square glass plate (1 x 1 cm) in a 24-well microplate and the AQs (56 μM) were added as described above. After the incubation period, the supernatants were removed, the biofilms were washed twice with sterile PBS and the glass plates were introduced vertically in the spectroscopic cuvettes at 45 degrees relative to the excitation light beam. Then the appropriate aqueous buffer (PBS or deuterated PBS) was carefully added to fill the cuvette. Controls containing sessile cells of *C*. *tropicalis* in D-PBS were performed. In agreement with the report by Berry *et al*. [30], D-PBS had no effect on the biofilm biomass compared to PBS. Spectroscopic measurements were carried out without stirring to prevent mechanically disturbing the biofilms.

### Effect of quenchers on the photo-induced biofilm reduction

Insight on the photo-induced biofilm reduction mechanism was gained by studying the effect of the ROS quenchers Tiron (for O_2_^•−^) and sodium azide (for ^1^O_2_) on a 48h-biofilm [31,32]. AQs (56 μM) and quenchers (500 mM) were added to biofilms at the same time, and the assay was performed in the dark and under irradiation using the conditions mentioned above. After 48 h incubation at 37°C, the supernatant was removed in order to measure the O_2_^•−^ production and the SOD activity. Biofilm formation was determined by CV, where BBU was calculated from OD measurements at 595 nm [13,14].

Production of O_2_^•−^ was evaluated by reduction of NBT (1 mg/mL) which converts in an insoluble precipitate (Blue diformazan) by action of this ROS, so that the formed blue diformazan is proportional to the generated O_2_^•−^ in biofilms, and was measured spectrophotometrically at 540 nm. Results were expressed as OD_540nm_/BBU (O_2_^•−^/BBU) [13,14].

Likewise, SOD activation was determined from the inhibition of NBT reduction by supernatant. O_2_^•−^ was generated by photoexcitation of riboflavin in the presence of oxygen and of the electron donor methionine. The results were expressed as SOD activation (%)/BBU [13,14].

### Statistical analysis

All assays were evaluated in triplicate and in 3 independent experiments. Results of all experiments were expressed as means values with standard deviations. Data were statistically analysed by using the Student-Newman-Keuls test for multiple comparisons and the post hoc Tukey’s test. A p-value < 0.05 was considered statistically significant.

## Results

### *In vitro* fungal biofilm reduction

**Fig 3:**
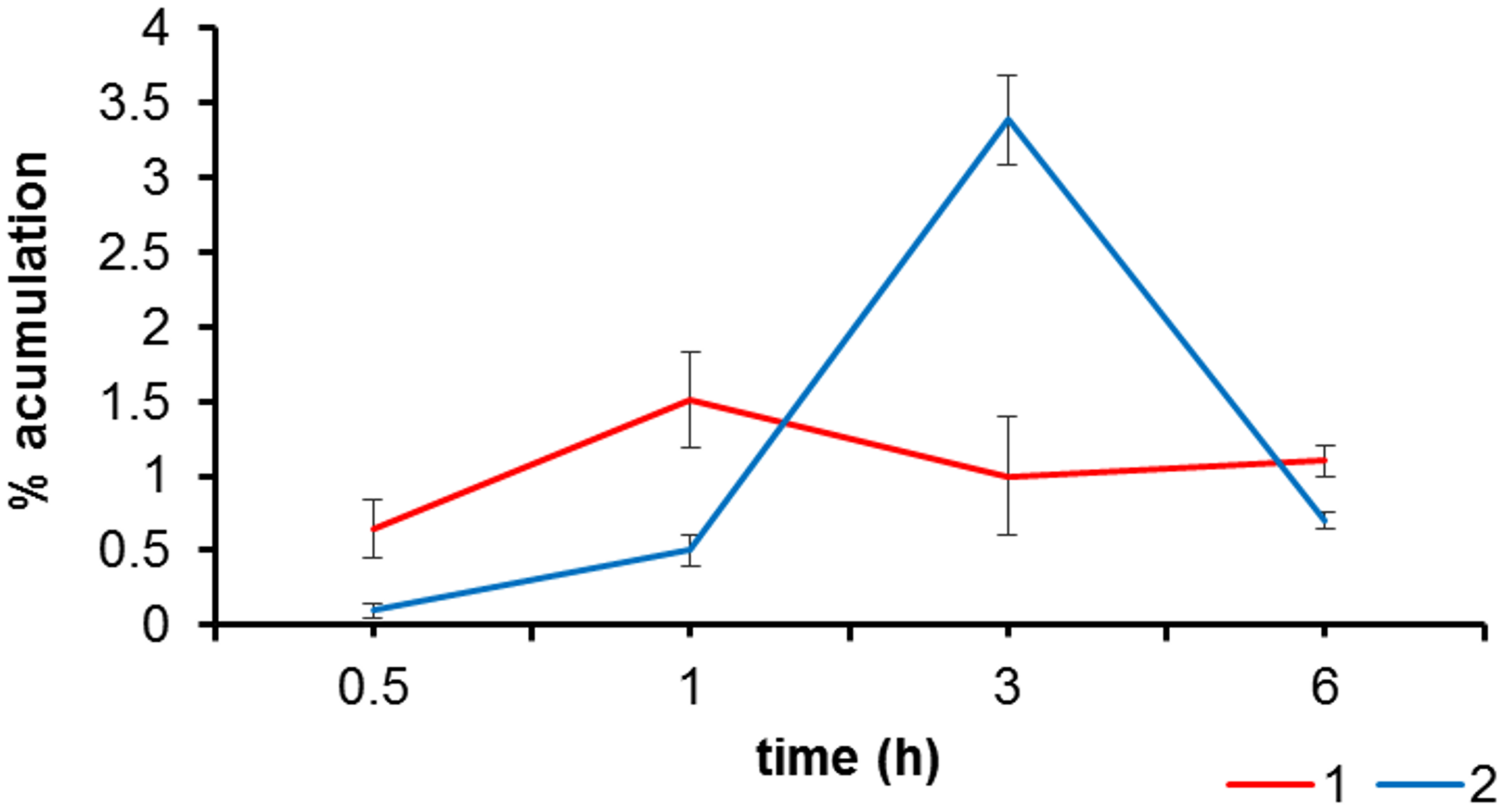
AQs accumulation in *C*. *tropicalis* biofilms by HPLC method.

AQ **2** accumulates in the biofilms to a substantially greater extent than **1**, (3.4 ± 0.3% *vs* 1.1 ± 0.4%, respectively), relative to the AQs initial concentration and after 3h incubation (Fig 3). When kept in the dark, neither **1** nor **2** were active against *C*. *tropicalis* NCFP biofilms at the concentration tested (56 μM) (Table 1). On the other hand, exposure to light reduced the BBU by 3.2 and 2.6-fold, respectively (p>0.05 when **1** is compared with **2**). It should be noted that light alone did not show any inhibitory effect on biofilm growth. Likewise, DMSO had no effect either.

**Table 1:** Mass of the biofilms (in BBU units) exposed to 56 μM rubiadin (1) or rubiadin 1-methyl ether (2) in the dark and under irradiation.

AQ	DARK	IRRADIATION
**1**	54 ± 3	18 ± 4*
**2**	50 ± 6	23 ± 8
Control**	57 ± 7	59 ± 5

*p>0.05 when **1** is compared with **2**

**No AQ added

### Absorption and fluorescence spectra

**Fig 4:**
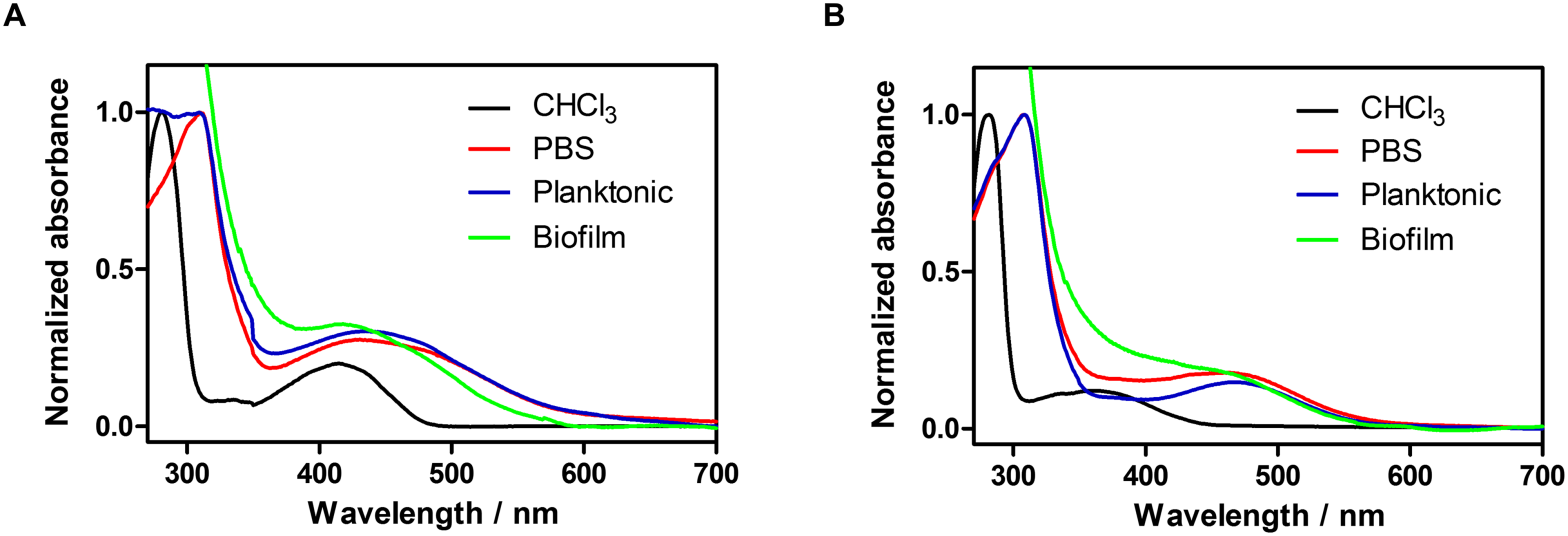
Absorption spectra of rubiadin (A) and rubiadin 1-methyl ether (B) (56 μM) in CHCl_3_, PBS, planktonic yeast and biofilms of *C*. *tropicalis*.

The absorption spectra of **1** and **2** in CHCl_3_, PBS, planktonic yeast and biofilms are shown in Fig 4. The spectra in PBS and planktonic yeast were similar, with broader, red-shifted bands relative to CHCl_3_ solutions. The red- shift is more pronounced for **2**. The spectra in biofilms showed a behaviour intermediate between CHCl_3_ and PBS.

Although the fluorescence was very weak, it was possible to determine the lifetime of the singlet excited states, which was 0.9 ns in CHCl_3_ and 0.60 ns in PBS for **1**. The corresponding values for **2** were 1.4 ns and 0.8 ns, respectively.

### Transient absorption experiments

**Fig 5:**
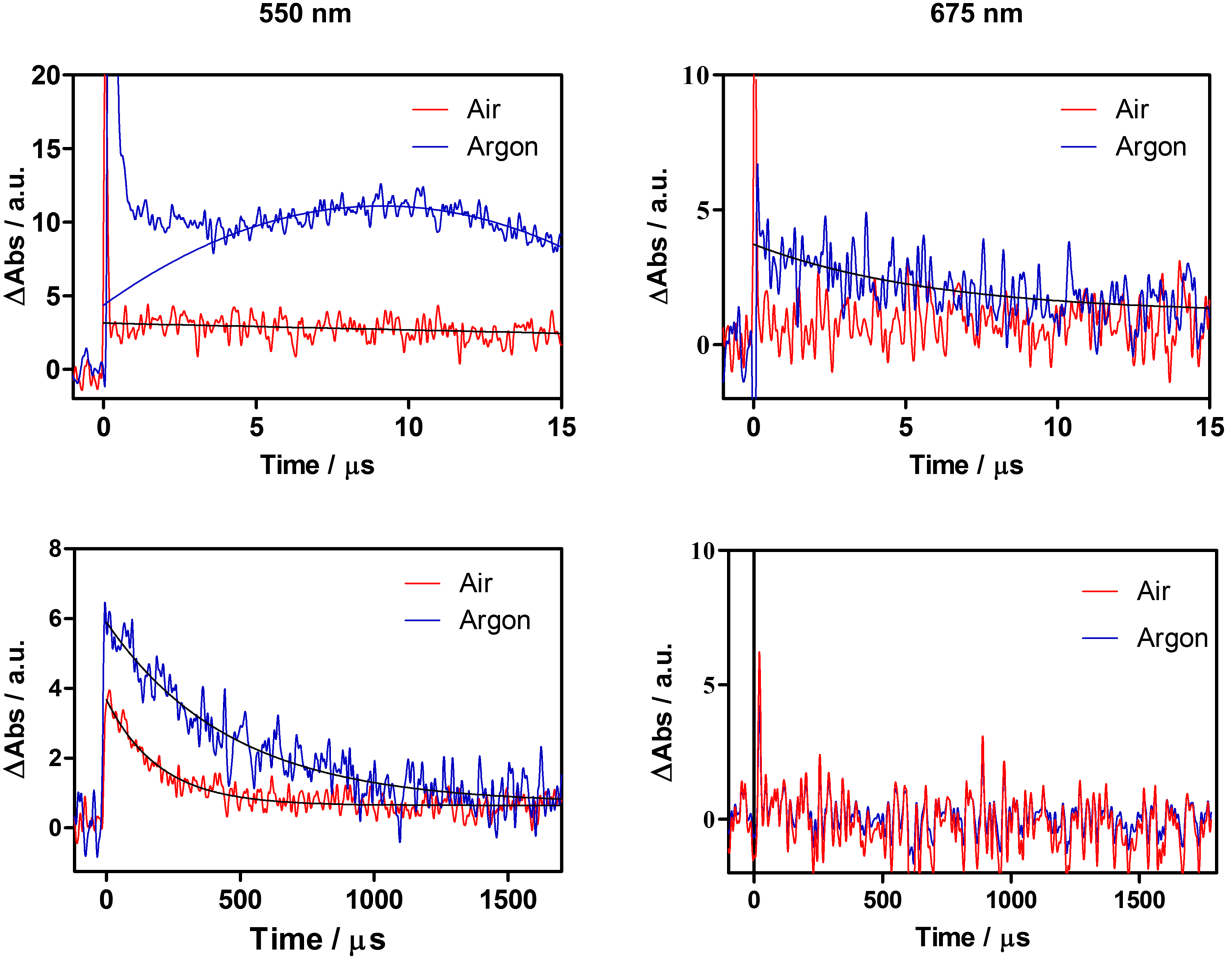
Transient absorption of 2 in argon- and air saturated- Cl_3_CH solutions. Signals recorded at 550 nm correspond to AQ^•−^, whereas signal at 675 nm correspond to ^3^AQ.

Formation of the triplet state, the ketyl radical and the radical anion of the AQs, ^3^AQ and AQ^•−^ respectively, was studied by transient absorption spectroscopy in CHCl_3_, PBS, planktonic yeast and biofilms. The corresponding wavelengths were 675 nm (^3^AQ,), 450 nm (AQ^•^) and 550 nm (AQ^•−^). It was not possible to detect AQ^•^ or AQ^•−^ for **1** in any of the above systems, whereas large AQ^•−^ signals could be observed for **2** in CHCl_3_ solutions (Fig 5). In argon-saturated CHCl_3_ the transient rose with lifetime 4 μs and lived 450 μs, whereas in air-saturated solutions the rise was much faster and the lifetime was decreased to 150 μs. This indicates that AQ^•−^ is formed from ^3^AQ and that oxygen quenches it, presumably leading to the production of O_2_^•−^ (Eq 1):

$${\text{AQ}}^{•}-+\;{\text{O}}_{2}\to \text{AQ }+{\text{O}}_{2}{}^{•}-$$ (1)

**Fig 6:**
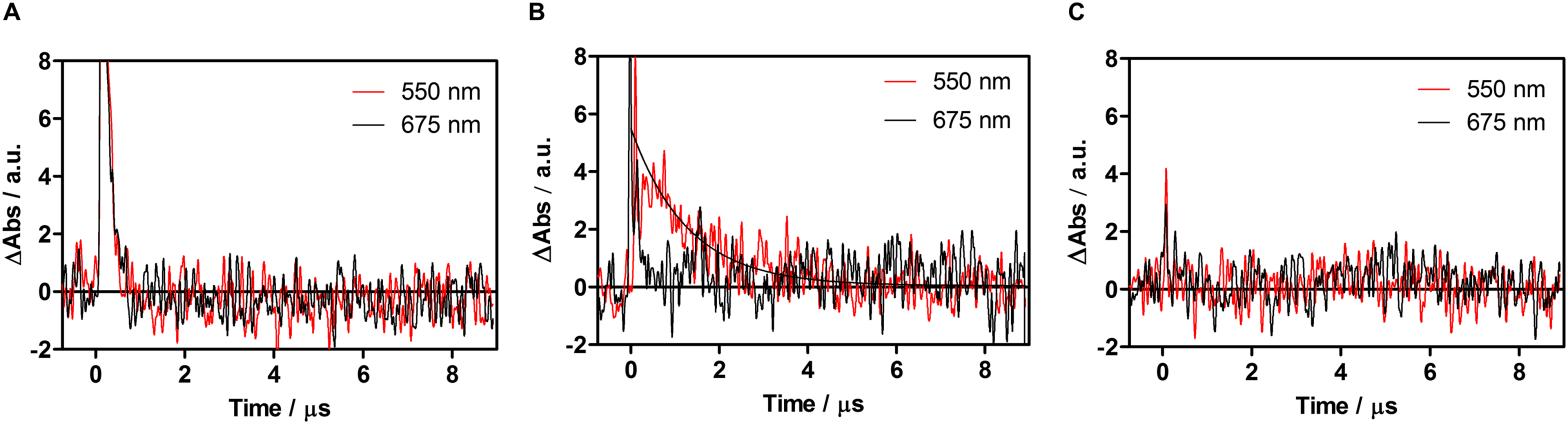
Transient absorption of air saturated- planktonic yeast suspension with 1 (A), 2 (B) and alone (C). Signals recorded at 550 nm correspond to AQ^•−^, whereas signal at 675 nm correspond to ^3^AQ.

**Fig 7:**
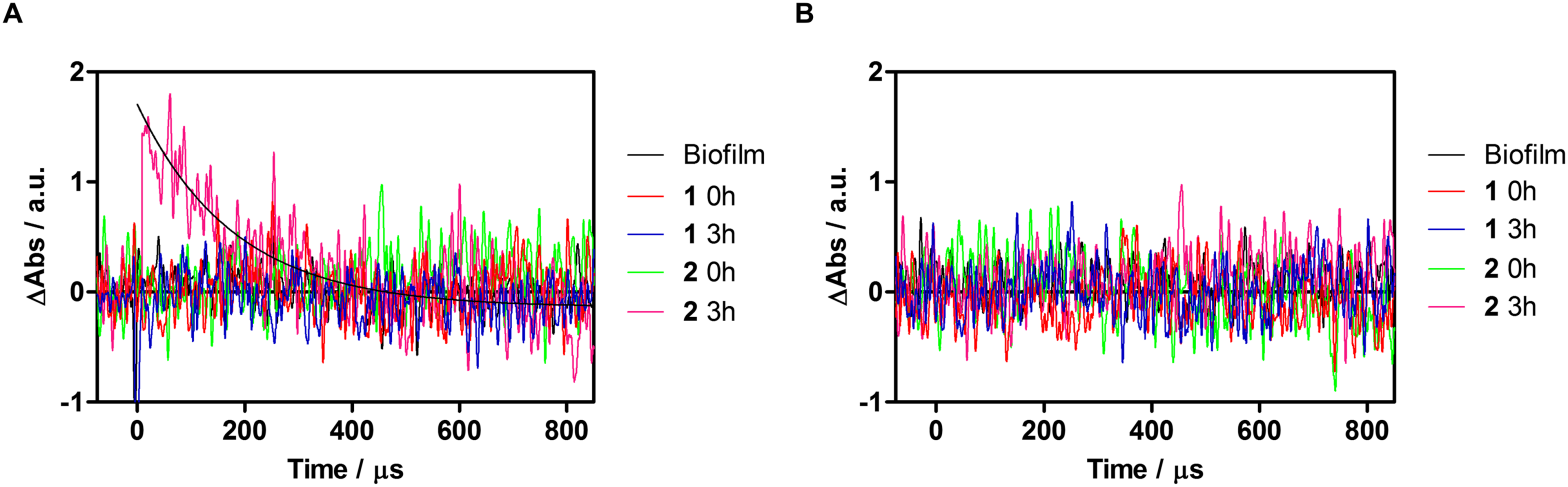
Transient absorption of air saturated- *C*. *tropicalis* biofilms with 1, 2 and alone at t = 0 and 3 hours of incubation. Signals recorded at 550 nm correspond to AQ^•−^(**A**), whereas signal at 675 nm correspond to ^3^AQ (**B**).

Consistent with the above observations, ^3^AQ was observed to decay with a similar lifetime, 5 μs, in argon-saturated solutions and it also disappeared when oxygen was allowed into the system. Weak transients could be observed in PBS living approximately 1 μs, however oxygen had no effect on their decay kinetics, neither was there any difference when was observed at 550 or 675 nm. They are thus tentatively attributed to aggregate species. A weak transient living 1.7 μs could be observed for AQ^•−^ (550 nm) in air-saturated planktonic yeast suspensions, whereas no signal was detectable at 675 nm (^3^AQ) (Fig 6B). In the case of biofilms, AQ^•−^ could be detected only when the cells were incubated for 3 h (Fig 7). The lifetime of the transient was 170 μs in air-saturated samples. No evidence for the presence of ^3^AQ in biofilms could be derived from transient absorption measurements (Fig 6).

### Singlet oxygen measurements

Time-resolved near-IR luminescence detection is a powerful tool to study the process of ^1^O_2_ photosensitization, since this ROS can be directly monitored through its phosphorescence at 1275 nm [26, 33–35]. Clear signals could be observed for **1** and **2** in CHCl_3_ and PBS. The Φ_Δ_ values in CHCl_3_ (0.34 and <10^−3^ for **1** and **2**, respectively) were already reported by Núñez Montoya et al. [15,16]. In PBS, they were Φ_Δ_ < 0.01 for **1** and Φ_Δ_ = 0.02 for **2**.

**Fig 8:**
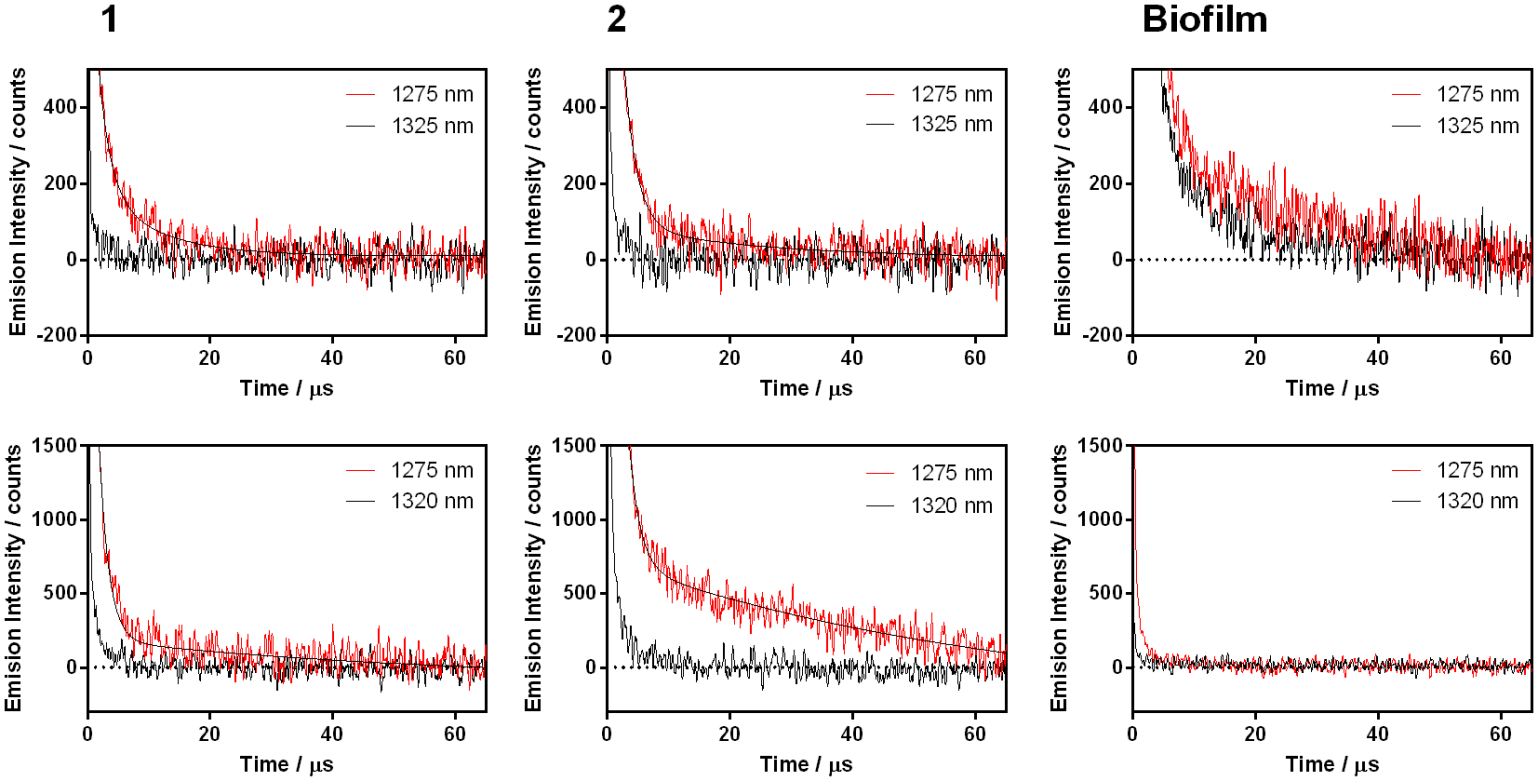
^1^O_2_ kinetics of 1 and 2 in *C*. *tropicalis* biofilm observed at 1275 nm and at 1325 nm (negative control) in PBS (A) and in biofilms incubated with D-PBS (B).

Luminescence signals could also be recorded in biofilms (Fig 8) and were assigned to ^1^O_2_ based on the spectral distribution (disappearance of the signal at 1325 nm, where ^1^O_2_ shows almost no phosphorescence), and on the lengthening of the decay lifetime upon solvent deuteration [34]. Observation of ^1^O_2_ indicates that ^3^AQ is indeed formed, even if the concentration is too small to produce a transient absorption signal. By comparing the intensity of the phosphorescence signals for the two AQs, it is apparent that **2** generates approximately three-fold more ^1^O_2_ than **1** in biofilms (Fig 8B). ^1^O_2_ production was not detected when the biofilm was incubated in PBS with either AQ (Fig 8A).

### Effect of ROS quenchers on the photo-induced biofilm reduction

**Fig 9:**
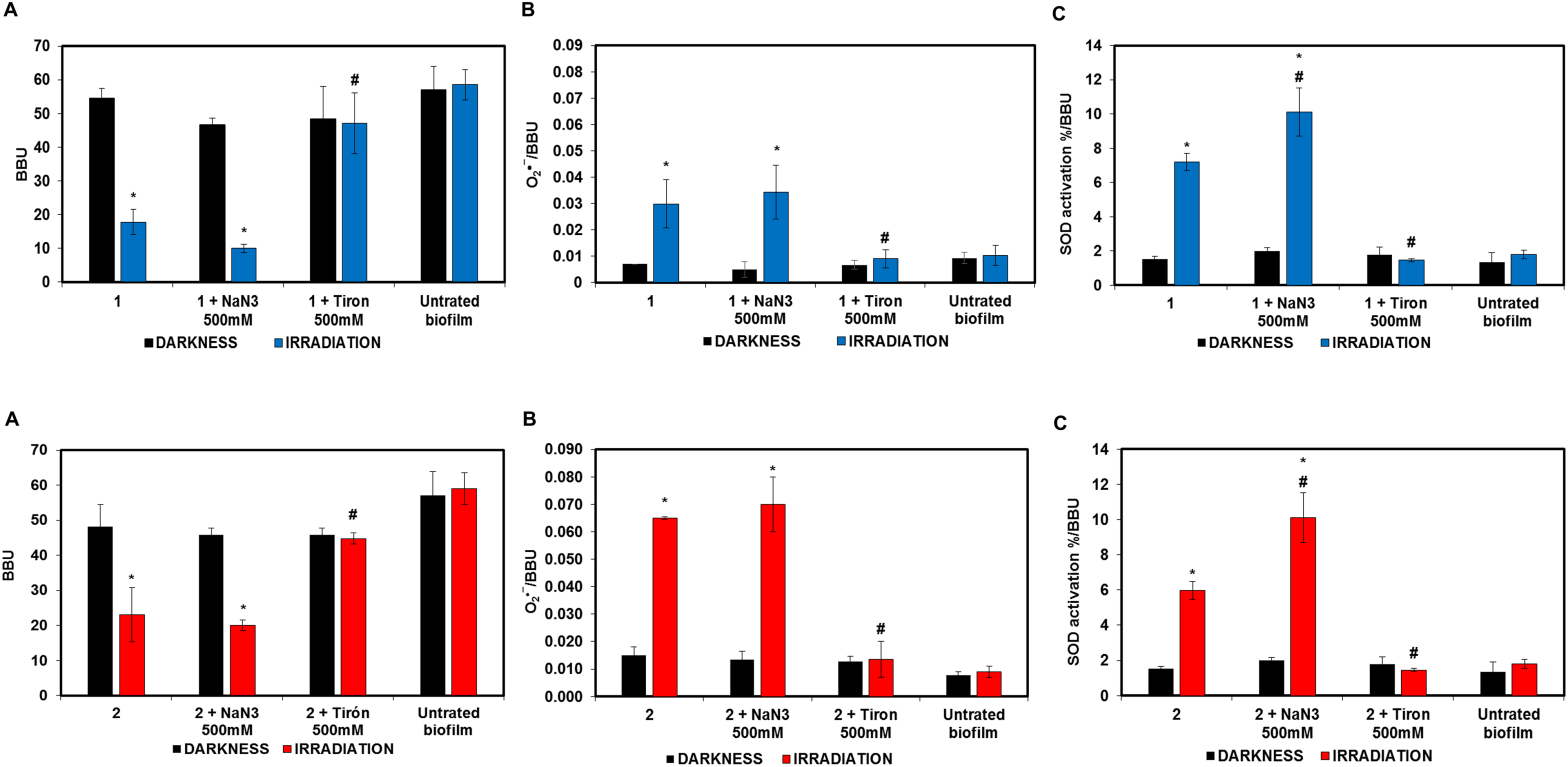
**(A) Photo-induced *C*. *tropicalis* biofilm reduction by the AQs 1 and 2 in the presence of the specific ROS quenchers sodium azide and Tiron. (B) Superoxide radical production in *C*. *tropicalis* by AQ alone and in the presence of quenchers. (C) SOD activation in *C*. *tropicalis biofilms* by AQ alone and in the presence of quenchers**.^*^ *p< 0*.*05* respect to biofilm ^#^ *p< 0*.*05* AQ *vs* AQ + Quencher.

The photo-induced biofilm reduction studies were repeated in the presence of specific ROS quenchers such as Tiron (for O_2_^•−^) and sodium azide (for ^1^O_2_). The photoinduced antibiofilm activity of **1** and **2** was not modified by sodium azide, whereas it was fully reversed by Tiron, reaching the control levels (biofilm) (Fig 9A). This implies that photostimulated AQs generate O_2_^•−^, however quenching by Tiron scavenges their antifungal activity. This was corroborated by the NBT assay (Fig 9B), which demonstrated that the anti-biofilm activity enhancement under irradiation correlates with increased levels of O_2_^•−^ and therefore with the enhanced activation of SOD (Fig 9C). Only in the presence of Tiron the production of O_2_^•−^ and the activation of SOD return to their basal levels (biofilm).

## Discussion

PDT of deeply-seated lesions uses red light owing to its higher penetration in tissues, however blue light is actually preferred for the treatment of surface infections and biofilms [36–39], which would be feasible with the current AQs. It is known that 410 nm blue light is capable of inactivating planktonic *Candida* spp. cells [40]. However, Fig 9A shows that the biomass of biofilms exposed to light without the AQs is not different from those with no drug and kept in the dark. The discrepancy is due the underlying reason may be that the light fluence used in that Zhang *et al*. study on planktonic cells [40], which was more than one order of magnitude larger than in the present work. While the AQs showed a similar antibiofilm effect under irradiation at the assayed concentration (56 μM ≈ 15 μg/mL), **2** was less cytotoxic than **1** on mammalian eukaryotic cells [24]. Specifically, **2** exhibited a CC_50_ value of 34.4 ± 0.2 μg/mL, whereas the CC_50_ value of 1 was 14.9 ± 0.2 μg/mL. It must be noted however that **1** showed a comparable antibiofilm activity already at 1.96 μg/mL.

Although the concentration of **1** was increased by one order of magnitude relative to that used in our previous publication [14], the activity on biofilms did not change appreciably. Specifically, a 63.5 ± 4.5% reduction was observed previously at 1.96 μg/mL (Table 1 in S1 File), whereas the currently observed decrease is 68.2 ± 6.7% at now at 14.22 μg/mL (56 μM) (p> 0.05). Thus, the antibiofilm effect of **1** does not change in this concentration range, which could be due to aggregation of **1** as indicated by the absorption spectra (Fig 4). The antibiofilm effect of **2** was similar to that previously observed, not surprisingly, because the concentration used was very similar.

The mechanism of photo-induced *C*. *tropicalis* biofilm reduction by two natural AQs, rubiadin and rubiadin-1-methyl ether, was studied as a means to identify the ROS involved in this photoprocess.

Absorption spectra (Fig 3) revealed the presence of AQ aggregates in PBS, planktonic yeast and biofilms, as indicated by the broadening of the bands and the changes in their relative intensity as compared to CHCl_3_ [41,42]. Nevertheless, the situation observed in biofilms suggests an intermediate behaviour in which partial disaggregation occurs upon biofilm uptake.

In one hand, although it was not possible to detect triplet state absorption for any of the AQs in biofilms, observation of ^1^O_2_ phosphorescence indicates that this species is actually formed. Once again, **2** shows a higher efficiency than **1**. There are no literature reports of the lifetime of ^1^O_2_ in *C*. *tropicalis* biofilms, therefore it is not possible to confirm whether the decays are characteristic or not. Thus, we had to rely on the accepted tests for ^1^O_2_ to ascertain the origin of the luminescence: spectral dependence on the phosphorescence intensity (higher at 1275 nm than at 1325 nm) and longer lifetime upon solvent deuteration. Biofilms incubated with **1** or **2** showed this pattern, more easily distinguishable in d-PBS incubated biofilms, confirming the assignment of the luminescence to ^1^O_2_ phosphorescence. There is also a strong component that dominates the early part of the decay, which is due to laser light scattering and is unavoidable in non-transparent samples. Nevertheless, the contribution of ^1^O_2_ to biofilm reduction must be negligible since sodium azide, a specific ^1^O_2_ quencher, was not able to prevent it.

Fig 9B shows that biofilms generate a non-zero level of O_2_^•−^ in the absence of light or AQ. The observation that incubation with AQs and exposure to light induced higher levels of O_2_^•−^ 48 h after the treatment (Fig 9B) indicates that the intrinsic ability of the cell to produce O_2_^•−^ had been perturbed. Since sodium azide did not quench the delayed production of O_2_^•−^ but Tiron did, it can be concluded that “prompt” O_2_^•−^ produced by photosensitization, but not ^1^O_2_, is the main responsible for the perturbation of the oxidative balance of the cell that leads to an increase in the “delayed” production of O_2_^•−^.

Tiron can react with both hydroxyl radicals (OH^•^) and O_2_^•−^ [43]. However, since Tiron was added before irradiation and OH^•^ is a secondary product of O_2_^•−^, Tiron would have prevented the formation of OH^•^ by scavenging its precursor, O_2_^•−^. On the other hand, the radical anion of AQ **2**, which is a precursor of O_2_^•−^ according to the reaction (1), was detected by transient absorption spectroscopy. Finally, ^1^O_2_ cannot be a precursor of O_2_^•−^, since azide, a well-known ^1^O_2_ quencher, did not prevent its production nor the growth of the biofilms.

In summary, the observation that Tiron inhibits the photo-induced *C*. *tropicalis* biofilm reduction by the AQs (Fig 9A) suggests that production of O_2_^•−^ by electron transfer quenching of the AQs excited states is the main component in the photosentizing mechanism.

## Conclusions

The results above indicate that the biomass reduction of *C*. *tropicalis* biofilms produced by the assayed natural AQs, Rubiadin and Rubiadin-1-methyl ether, was mediated mainly by O_2_^•−^ generation after exposure to light (Type-I photodynamic mechanism). Production of ^1^O_2_ was observed in biofilm incubated with deuterated PBS, however its participation in the photo-induced biofilm reduction seems negligible.

## Supporting information

S1 File Marioni et al 2016-AQs biofilms. AQs reduce *Candida tropicalis* biofilms.
(PDF)
