# Supplementary material for: On the mechanism of Candida tropicalis biofilm reduction by the combined action of naturally-occurring anthraquinones and blue light
Source: PLoS One. 2017 Jul 19;12(7):e0181517. doi: 10.1371/journal.pone.0181517 (PMC5517063; doi:10.1371/journal.pone.0181517)
Supplement: S1 File — AQs reduce Candida tropicalis biofilms. (PDF) [file pone.0181517.s001.pdf]

Phytomedicine 23 (2016) 1321–1328

Contents lists available at ScienceDirect

Phytomedicine

journal homepage: www.elsevier.com/locate/phymed

The anthraquinones rubiadin and its 1-methyl ether isolated from *Heterophyllaea pustulata* reduces *Candida tropicalis* biofilms formation

Juliana Marioni[a], María Angel da Silva[b], José Luis Cabrera[a], Susana C. Núñez Montoya[a], María Gabriela Paraje[b,*]

[a] Instituto Multidisciplinario de Biología Vegetal (IMBIV) - Consejo Nacional de Investigaciones Científicas y Técnicas (CONICET), Departamento de Farmacia, Facultad de Ciencias Químicas, Universidad Nacional de Córdoba, Argentina. Haya de la Torre y Medina Allende, X5000HUA Córdoba, Argentina

[b] Instituto Multidisciplinario de Biología Vegetal (IMBIV) - Consejo Nacional de Investigaciones Científicas y Técnicas (CONICET), Cátedra de Microbiología, Facultad de Ciencias Exactas Físicas y Naturales. Universidad Nacional de Córdoba, Argentina. Av. Vélez Sarsfield 299, Córdoba, Argentina

ARTICLE INFO

Article history:
Received 12 February 2016
Revised 5 July 2016
Accepted 19 July 2016

Keywords:
Heterophyllaea pustulata
Candida tropicalis
Biofilms
Antifungal
Oxidative stress
Synergistic activity

ABSTRACT

Background: *Candida tropicalis* is increasingly becoming among the most commonly isolated pathogens causing fungal infections with an important biofilm-forming capacity.
Purpose: This study addresses the antifungal effect of rubiadin (AQ1) and rubiadin 1-methyl ether (AQ2), two photosensitizing anthraquinones (AQs) isolated from *Heterophyllaea pustulata*, against *C. tropicalis* biofilms, by studying the cellular stress and antioxidant response in two experimental conditions: darkness and irradiation. The combination with Amphotericin B (AmB) was assayed to evaluate the synergic effect.
Study design/Methods: Biofilms of clinical isolates and reference strain of *Candida tropicalis* were treated with AQs (AQ1 or AQ2) and/or AmB, and the biofilms depletion was studied by crystal violet and confocal scanning laser microscopy (CSLM). The oxidant metabolites production and the response of antioxidant defense system were also evaluated under dark and irradiation conditions, being the light a trigger for photo-activation of the AQs. The Reactive Oxygen Species (ROS) were detected by the reduction of Nitro Blue Tetrazolium test, and Reactive Nitrogen Intermediates (RNI) by the Griess assay. ROS accumulation was also detected inside biofilms by using 2′,7′-dichlorodihydrofluorescein diacetate (DCFH-DA) probe, which was visualized by CSLM. Superoxide dismutase (SOD) activity and the total antioxidant capacity of biofilms were measured by spectrophotometric methods.

The minimun inhibitory concentration for sessile cells (SMIC) was determined for each AQs and AmB. The fractional inhibitory concentration index (FICI) was calculated for the combinations of each AQ with AmB by the checkerboard microdilution method.
Results: Biofilm reduction of both strains was more effective with AQ1 than with AQ2. The antifungal effect was mediated by an oxidative and nitrosative stress under irradiation, with a significant accumulation of endogenous ROS detected by CSLM and an increase in the SOD activity. Thus, the prooxidant-antioxidant balance was altered especially by AQ1. The best synergic combination with AmB was also obtained with AQ1 (80.5%) (FICI = 0.74).
Conclusion: Under irradiation, the oxidative stress was the predominant effect, altering the prooxidant-antioxidant balance, which may be the cause of the irreversible cell injury in the biofilm. Our results showed synergism of these natural AQs with AmB. Therefore, the photosensitizing AQ1 could be an alternative for the *Candida* infections treatment, which deserves further investigation.

© 2016 Elsevier GmbH. All rights reserved.

Abbreviations: DCFH, 2′,7′-dichlorodihydrofluorescein; DCFH-DA, 2′,7′-dichlorodihydrofluorescein diacetate; DCF, 2′,7′-dichlorofluorescein; AmB, Amphotericin B; AQs, Anthraquinones; AQ1, Rubiadin; AQ2, Rubiadin 1-methyl ether; BBU, Biofilm Biomass Unit; CLSI, Clinical and Laboratory Standards Institute; CFU, Colony forming unit; CSLM, Confocal scanning laser microscopy; CV, Crystal violet; FRAP, Ferrous reduction antioxidant potency assay; FIC, Fractional inhibitory concentration; FICI, Fractional inhibitory concentration index; H_2O_2, Hydrogen peroxide; MIC, Minimum inhibitory concentration; NO, Nitric Oxide; NBT, Nitro blue tetrazolium; OD, Optical density; PBS, Phosphate-buffered saline; RNI, Reactive nitrogen intermediates; ROS, Reactive oxygen species; SDB, Sabouraud Dextrose Broth; SDA, Sabouraud dextrose agar; SMIC, Sessile minimum inhibitory concentration; SMIC 50, Sessile minimum inhibitory concentration 50; SMIC 80, Sessile minimum inhibitory concentration 80; Sub MIC, Sub Minimum Inhibitory Concentration; SOD, Superoxide dismutase; $O_2^{\bullet -}$, Superoxide radical; Supra MIC, Supra Minimum Inhibitory Concentration.

* Corresponding author. Fax: +54 351 4334198.
E-mail address: gabrielaparaje@gmail.com, paraje@efn.uncor.edu (M.G. Paraje).

http://dx.doi.org/10.1016/j.phymed.2016.07.008
0944-7113/© 2016 Elsevier GmbH. All rights reserved.

Introduction

Candida albicans is the most prevalent cause of fungal infections in humans, with more than 50% of mucocutaneous and systemic yeast infections (Pfaller, 2012). Nevertheless, *Non-albicans Candida* species is increasingly becoming more relevant. For instance, the incidence of fungal infections by *C. glabrata, C. parapsilosis* and *C. tropicalis* has increased in recent years (Raman et al., 2015). *Candida* species are known to *develop* several mechanisms that confer resistance to antifungal drugs, which are well described and characterized for planktonic (or free-living) cells. However, resilient infections are invariably associated with biofilms development, since this kind of growth exhibits a dramatic decrease in the susceptibility to the antimicrobial agents. Therefore, the biofilm formation is considered an important virulence factor that is frequently associated with clinical infections generated by its development in various invasive and indwelling medical devices, such as central venous catheters, joint prostheses, cardiovascular devices, as well as the superficial devices like dentures and dental implants (Uppuluri et al., 2009; Silva-Dias et al., 2015).

The rate of drugs diffusion in *C. tropicalis* biofilms is lower than in biofilms of *C. glabrata* or *C. krusei* (Al-Fattani and Douglas, 2006). This behavior is an answer to the particular characteristic of the *C. tropicalis* biofilms that synthetize large amounts of hexosamine-enriched matrix, so that not only the quantity but also the matrix composition allow a poor penetration by antifungal agents; and thus these biofilms are more resistant to Amphotericin B (AmB) (Uppuluri et al., 2009; Ramage et al., 2012). On the other hand, antifungal drugs available to successfully treat systemic and invasive candidiasis are becoming increasingly limited (Tobudic et al., 2012). Therefore, there is a growing medical need for new agents and therapeutic strategies to treat candidiasis. The development of novel approaches to inactivate *Candida* biofilms has a great clinical importance in treating this infection, among which can be mentioned the combination therapy that has the advantage of attacking different targets by the combination of several drugs and/or strategies with different action mechanisms.

There is an increasing evidence that the mechanism of cell death initiated by some antifungal involves the production of Reactive Oxygen Species (ROS) (Delattin et al., 2014), having been reported that miconazole induces the accumulation of endogenous ROS in *C. albicans* biofilms (Vandenbosch et al., 2010). Nevertheless, a high ROS-detoxifying activity by superoxide dismutase enzyme (SOD) has been determined to this kind of drugs. These enzymes appear to play an important role in protecting *C. albicans* biofilms against high doses of miconazole through a fungal biofilm resistance mechanism (Mah, 2012).

In addition, we previously reported that ROS, Reactive Nitrogen Intermediates (RNI) and their downstream derivatives could play an important role on the biofilm formation (Arce Miranda et al., 2011; Peralta et al., 2015). The ROS and RNI overproduction, favored by some conditions, results in a cellular stress inside the biofilms, thereby affecting their growth. Specifically, it was observed an extracellular matrix reduction because of an accumulation of these radical oxidizers in the extracellular medium, and thus they affect the matrix (Arce Miranda et al., 2011). Redox imbalance is due to an overproduction of ROS or through a reduction in the oxidative defenses being insufficient to remove the free radicals, and therefore the antioxidant system plays a very important role in the control of this process. (Berg et al, 2004).

Our research group has isolated several 9,10-anthraquinone aglycones (AQs) from a phototoxic plant popularly known as "cegadera", name that alludes to one of the toxic effects produced in cattle that feeds on it (Núñez Montoya et al., 2003). This vegetal species, scientifically identified as *Heterophyllaea pustulata* Hook f. (Rubiaceae), is endemic to the mountain region of northwestern

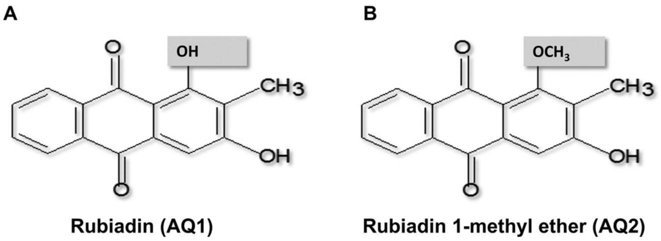

Fig. 1. (A) Structure of Rubiadin (AQ1) and (B) Rubiadin 1-methyl ether (AQ2) isolated from *Heterophyllaea pustulata* Hook f. (Rubiaceae).

Argentina and Bolivia, between 2500 and 3000 m of altitude. In addition, we have demonstrated that these AQs possessed photosensitizing properties; hence they generate ROS under light action (Núñez Montoya et al., 2005).

The present work was performed with the aim of knowing the potential antibiofilm effect of two photosensitizing AQs from *H. pustulata*: rubiadin (AQ1) and rubiadin 1-methyl ether (AQ2) (Fig. 1A and B), against sessile cells of *C. tropicalis*, and also evaluating if there is a disturbance of the prooxidant-antioxidant balance. Therefore, the production of oxidative and nitrosative metabolites, and the activation of antioxidant enzyme SOD and the total antioxidant capacity of the system were evaluated. In addition, the light action was specifically assessed as a trigger to increase the biological effect of these AQs. Consequently, AQ1 or AQ2 were also used to examine the antifungal effects of their combinations with AmB against *C. tropicalis* biofilms under irradiation. To our knowledge, this is the first study that attempts to correlate the biofilm reduction with alteration in the ROS and RNI production by the action of AQs under irradiation. Thus, the light action in the presence of oxygen improved the antimicrobial effect of photosensitizing anthraquinones by increasing the Redox imbalance, because of their ability to generate ROS, which resulted ultimately in an oxidative stress inside of *C. tropicalis* biofilm.

Material and methods

Extraction and isolation of AQs

AQ1 and AQ2 were isolated and purified from benzene extracts of roots of *H. pustulata* by following the methodology described by our researcher group (Núñez Montoya et al., 2003), and their structures were characterized by their spectroscopic/spectrometric data (^{1}H NMR, ^{13}C NMR, IR, UV–Vis, MS). The purity of AQ1 and AQ2 were 93.6 ± 0.1% and 93.8 ± 0.1% respectively, determined by HPLC and HPLC-SM analysis (Fig. 2A and B). A Varian Pro Star chromatography apparatus (model 210, series 04171), equipped with an UV–Vis detector was used. The separation was achieved on a Microsorb-MV column 100-5 C-18 (250 × 4.6 mm i.d., Agilent), at 25°C. The mobile phase consisted in formic acid (0.16 M, solvent A) and MeOH-formic acid (0.16 M, solvent B), starting with 48% B (2 min, 1.0 ml/min) that changed during 4 min to 78% and 0.8 ml/min (2 min), followed by a second ramp (2 min) to 84% B and 0.5 ml/min (30 min), a third ramp (1 min) to 100% B and 0.7 ml/min (4 min), returning to the starting conditions for 1 min. Detector was set at 269 nm. The manual injection volume was 20 µl. Data analysis was performed using Varian software (Star Chromatography Workstation 6.41).

Fungal strains and culture conditions

C. tropicalis biofilms were studied from a clinical isolate and a reference strain. The clinical isolate associated with indwelling medical devices (CRF2012, strain No. 1) was kindly identified by

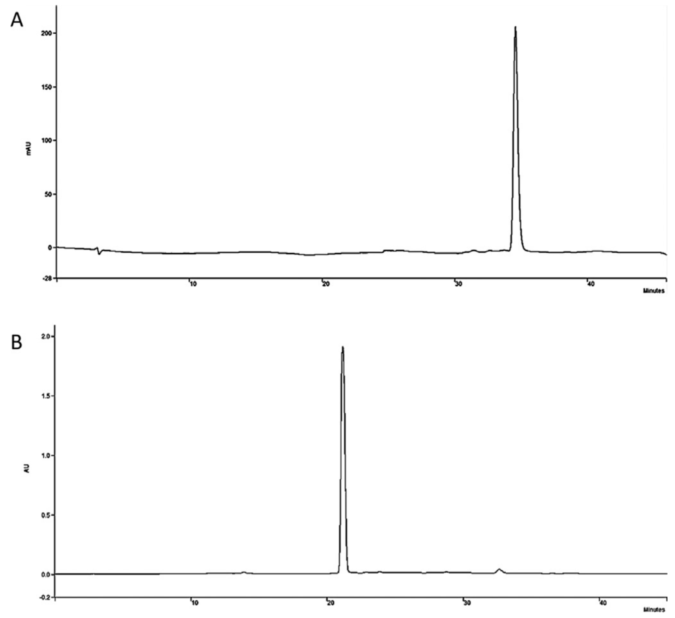

Fig. 2. HPLC chromatograms profiles showing the purity of AQ1 (A) and AQ2 (B).

the Microbiology Laboratory of Clínica Universitaria Reina Fabiola (Córdoba, Argentina). The reference strain was *C. tropicalis* NCPF 3111 (National Collection of Pathogenic Fungi, Bristol, UK, strain No. 2). For long-term storage, the Instituto Multidisciplinario de Biología Vegetal (IMBIV), yeasts stocks were kept at −80 °C suspended in Sabouraud Dextrose Broth (SDB) (Difco, Detroit, MI) with 10% glycerol as cryoprotectant. Before use, yeasts were plated onto Sabouraud dextrose agar (SDA) (Difco, Detroit, MI) and incubated overnight at 37 °C to ensure purity and viability (Peralta et al., 2015).

Quantification of biofilm formation

Biofilms were prepared in flat-bottomed 96-well microplates (Greiner Bio-One, Germany) by an adaptation from the method of O' Toole and Kolter (1998). Plates were pre-treated with 50% (v/v) fetal bovine serum (Sigma-Aldrich, St. Louis, MO, USA) (Pierce et al., 2008). A suspension (1×10^7 cells/ml) in SDB was inoculated and plates were incubated at 37 °C for 90 min. Non-adhered cells were removed and plates were incubated at 37 °C for 48 h without shaking. *C. tropicalis* biofilms were stained with crystal violet (CV) dye, which measures the total biomass of biofilm (Messier et al., 2011), by using a solution 1% (w/v) for 5 min and washing with sterile Phosphate-buffered saline (PBS) to remove excess dye. Afterwards, CV was extracted with the destaining solution: ethanol/acetone (70:30). Optical density (OD) was quantified at 595 nm using a microplate reader (Tecan Sunrise Model, TECAN, AUS). The average OD from control wells (ODc, containing only SDB at pH = 6.5) was subtracted from OD of all tested wells.

Strains were classified as biofilm producers according to the following classification: OD ≤ ODc = no biofilm producer; ODc < OD ≤ (2 × ODc) = weak biofilm producer; (2 × ODc) < OD ≤ (4 × ODc) = moderate biofilm producer; and (4 × ODc) < OD = strong biofilm producer. Biofilm Biomass Unit (BBU) was arbitrarily defined as 0.1 OD_{595} equal to 1 BBU (Arce Miranda et al., 2011; Peralta et al., 2015).

Antifungal activity

Antifungal activity of AQs and/or AmB against planktonic *C. tropicalis* was measured by quantifying the minimum inhibitory concentration (MIC) following the guidelines of the M27-A3 document by the Clinical Laboratory Standards Institute (CLSI, 2008). MIC was defined as the lowest drug concentration able to produce a growth inhibition higher than 90% in planktonic cells.

Effects of AQs over *C. tropicalis* biofilms were evaluated and AmB was used as positive control at the same conditions. Briefly, three dilutions of AQs (in SDB with 1% DMSO) were prepared in order to obtain final concentrations of 2 × MIC (Supra Minimum Inhibitory Concentration -Supra MIC), 1 × MIC, and 0.5 × MIC (Sub Minimum Inhibitory Concentration -Sub MIC). AQs or AmB (200 µl per well) solutions were added over 48 h-biofilms (each concentration in triplicate), and incubated 48 h at 37 °C. Negative controls were included, containing SDB alone or SDB with 1% DMSO. After incubation, supernatant was separated to assess oxidative metabolites and antioxidant activity of biofilm, and biofilm formation was then quantified as described previously. From this assay, sessile minimum inhibitory concentrations 50 and 80 (SMIC 50 and SMIC 80, respectively), defined as the drug concentrations which decrease 50 and 80% the BBU (Pierce et al., 2008), were obtained.

Two microplates were performed simultaneously under darkness and irradiation during 15 min with an TL 20 W/52 Phillips actinic lamp (380–480 nm, 0.65 mW/cm^2) with an emission maximum at 420 nm and located at 20 cm above the samples in a black box (Comini et al., 2011).

Colony forming units (CFU) /ml were determined for correlation studies with BBU (Peralta et al, 2015). After antifungal treatment, the supernatant was eliminated and 100 µl of sterile water was added to each well and sonicated (40 kHz, 60 s) in order to re-suspend the biofilm cells thoroughly. This suspension was diluted 1000 times with sterile water and 100 µl of the suspension was then pipeted out and spread evenly by using a sterile plastic transferring loop on SDA (Difco). Then the plates were incubated at 37 °C for 24 h.

Assays for oxidative metabolites and antioxidant activity of biofilms

Superoxide radical ($O_2^{\bullet -}$) was detected by their oxidative action that causes the nitro blue tetrazolium (100 µl of NBT 1 mg/ml, Sigma-Aldrich) reduction to nitroblue diformazan, by following the methodology previously described by us (Peralta et al., 2015; Marioni et al., 2016). NBT forms an insoluble dark blue diformazan precipitate, being proportional to the generated ROS in biofilms. Absorbance was measured spectrophotometrically at 540 nm Hydrogen peroxide (H_2O_2) treated samples were used as positive control. Results were expressed as $OD_{540\ nm}$/BBU (ROS/BBU) (Arce Miranda et al., 2011; Angel Villegas et al., 2015; Peralta et al., 2015).

Nitric oxide (NO) production was determined by measuring the accumulation of its stable degradation products, nitrate and nitrite, by a micro-plate assay using the Griess reagent and $NaNO_2$ as standard. Supernatant (100 µl) was mixed with 200 µl of Griess reagent [sulfanilamide 1.5% in 1 N HCL and N-1-naphthyl ethylenediamide dihidrochloride 0.13% in sterile distilled water] (Baronetti et al., 2011). OD was measured at 540 nm in the same microplate reader before mentioned. Results were expressed as the nitrite concentration value/BBU (RNI/BBU).

SOD activity was determined based on inhibition of NBT reduction by using 50 µl of supernatant. SOD inhibit the reduction of NBT by the action of the $O_2^{\bullet -}$, generated by the illumination of riboflavin. OD was measured at 595 nm, and the results were expressed as SOD activation (%)/BBU (Angel Villegas et al., 2015; Peralta et al., 2015).

Total antioxidant capacity of biofilm was evaluated by the ferric reducing antioxidant potency (FRAP) assay. Briefly, supernatant (10 µl) were mixed with 300 µl of the following mixture (10:1:1): (a) 300 mM acetate buffer pH: 3.6, (b) 10 mM 2,4,6-tripyridyl-s-triazine in 40 mM HCl and (c) 20 mM $FeCl_3 . 6H_2O$. OD was

measured at 593 nm after 4 min incubation, and FRAP values were calculated using a $FeSO_4$ calibration curve. Results were expressed as the Fe^{+2} concentration values/BBU (FRAP/BBU) (Peralta et al., 2015).

Biofilm analysis by confocal scanning laser microscopy (CSLM)

C. tropicalis biofilms treated with 1.96 µg/ml of AQs were observed by CSLM as described below. Biofilms were formed on small glass covers (12 mm diameter) in a 24-well microtiter plate (Greiner Bio-One, Germany). Following biofilm formation and antifungal exposure, supernatants were eliminated and disks were rinsed with sterile PBS (no autofluorescence detected). At first, disks were stained for 1 min with 30 µl of Calcofluor-White (0.05% v/v, Sigma-Aldrich), a carbohydrate-binding fluorescent dye that stains fungal cell walls blue. Calcofluor-White was excited at 355 nm (Peralta et al., 2015).

Intracellular ROS production was also determined by using a probe of the non-fluorescent and cell-permeating compound: 2′,7′-dichlorodihydrofluorescein diacetate (DCFH-DA; Molecular Probes, Inc., Eugene, Oreg.). After being washed in PBS, disks were incubated with 50 µl DCFH-DA (10 µM) for 15 min in darkness at room temperature. A 488 nm excitation source was used (Peralta et al., 2015). DCFH-DA is a non-polar, non-fluorescent compound that readily diffuses across membranes, and is hydrolyzed by intracellular esterases to the polar, non-fluorescent, membrane-impermeable derivative 2′,7′-dichlorodihydrofluorescein (DCFH), which is rapidly oxidized by ROS to the highly fluorescent 2′,7′-dichlorofluorescein (DCF) which is trapped within the cell (Bergamo et al., 2015).

After staining, disks were removed from the wells; air-dried for 15 min in darkness and placed inverted in 35-mm glass-bottom microwell dishes. Intact biofilms were examined by using a Fluoview FV1000 Espectral Olympus CSLM (Olympus Latin America, Miami, FL, USA) equipped with UPlanSApo 100X/1.40 oil UIS2 Olympus oil immersion lens. Optical sections were acquired at 0.5 µm intervals for the total thickness of biofilms. Then, for each sample, images from three randomly selected positions were obtained and analyzed independently. The quantitative analysis of DCF (green) fluorescence intensities was performed by means of the NIH-ImageJ.

Checkerboard microdilution assay

Checkerboard microdilution method was performed in flat-bottomed 96-well microplates. After the MICs of each drug for each strain were determined. The antifungal agents solution of AQs and AmB were used (2 × MIC, 1 × MIC, 0.5 × MIC). AmB solution was added by columns of the microplates, whereas AQs were added by rows over 48 h-biofilms and was incubated 48 h at 37 °C.

The fractional inhibitory concentration index (FICI) values were calculated as follows: FICI = SMIC50 (AB)/SMIC50 (A) + SMIC50 (AB)/SMIC50 (B). The SMIC (AB) represents the SMIC value of AQ1 or AQ2 combined with AmB, whereas SMIC (A) represents the SMIC values of AQ1 or AQ2 alone and SMIC (B) is the SMIC value of AmB. The FICI values were interpreted as follows: ≤0.5, synergy; >0.5 to <1, partial synergy; 1, addition; >1 to <4, indifference; and ≥4, antagonism (Dastgheyb et al., 2013).

Statistical analysis

All assays were performed in triplicate and in three independent experiments, and the averages and standard deviations were calculated for all of them. Numerical data were presented as means ± standard deviation. Differences between means were assessed using ANOVA followed by Student–Newman Keuls test for multiple comparisons. A $^*p < 0.005$ was considered significant for comparisons with non-treated biofilms and $^{\#}p < 0.005$ for comparisons between dark and light conditions.

Results

In order to be able to perform the antibiofilm experiments, the MICs values of AQs against planktonic fungal cells must be determined at first. The values obtained were: 0.98 µg/ml for AQ1 in both *C. tropicalis* strains (clinical strain, No. 1 and NCPF 3111 strain, No. 2); and 31.3 and 15.6 µg/ml for AQ2 in strain No. 1 and 2, respectively, clearly showing that AQ1 has better activity than AQ2 against both strains.

The ability of *C. tropicalis* strains to form biofilms was investigated on polystyrene-microtiter plates and were classified as strong biofilm producer (Table 1). BBU were not affected by light action. In darkness, AQ1 and AQ2 were not active against biofilms of both strains at all concentrations tested. On the other hand, the light action enhanced the effect of both AQs over biofilms of both strains. In strain No. 1, AQ1 and AQ2 reduced about 3.6 and 4.5 times the BBU at the MIC concentration, respectively. Furthermore, both AQs achieved the best reduction (5.6 times), at the SupraMIC. By contrast, biofilm reduction was lowest in strain No. 2 for both AQs at the MIC: BBU was reduced 1.8 times by AQ1, and 2.0 times by AQ2. These results demonstrate that AQ1 is more active than AQ2 in biofilms as well as in planktonic microorganism, since AQ1 required a smaller concentration than AQ2 to produce a similar reduction level. A correlation between CV assay and CFU/ml was observed (see in supporting information). Furthermore, when evaluating sessile cells, the antifungal concentration required to reducing the BBU to 50% (SMIC 50) was 1.96 µg/ml for AQ1 against strain No. 2. This effect was greatest on biofilms of strain No. 1, achieving an 80% reduction at the same concentration (1.96 µg/ml = SMIC 80).

In order to determine if cellular stress is implied on the AQ1 mechanism by light action, $O_2^{\bullet -}$ production was determined by the NBT assay (Fig. 3A). $O_2^{\bullet -}$ production was increased significantly in both strains under irradiation ($^{\#}p < 0.005$). The highest ROS production was obtained at Supra MIC of AQ1 compared to untreated biofilm ($^*p < 0.005$) and the AQ1 control did not produce $O_2^{\bullet -}$ in the presence of light by itself. Since the RNI have shown to promote cell death and biofilms dispersal, the RNI generation was measured. Fig. 3B shows an increase of RNI levels in strain No. 1 biofilm by AQ1, under both experimental conditions relative to control ($^*p < 0.005$). It can be seen that the RNI levels were proportionally dependent on AQ1 concentrations. Although a RNI increase was observed under darkness and irradiation, in the latter, the increase was more significant ($^{\#}p < 0.005$). On the other hand, in strain No. 2, the RNI were only increased by the light action at the MIC and Supra MIC concentrations of AQ1, showing a good correlation with results displayed in Table 1 (Fig. 3B).

A fluorogenic dye (DCFH-DA) was used to determine the intracellular ROS generation inside *C. tropicalis* biofilms after antifungal treatment. The laser scanning fluorescence images for XY (top) and XZ (bottom) of sessile cells (blue) of *C. tropicalis* NCPF 3111 biofilms showed a hazy biofilm appearance due to the diffuse staining of the extracellular material with Calcofluor-White, which implies that this material is mainly composed of cell-wall-like polysaccharides (Fig. 4A and D). Moreover, biofilm thickness was reduced by AQ1 action, corresponding to a 68.8% compared to respective control. ROS production was observed inside sessile cells, resulting in a high-intensity DCF fluorescence for the biofilm treated with AQ1 compared with untreated (control) (Fig. 4B and E). Co-localization is observed in Fig. 4C and F as a different additive color when the images were colorized and merged into a single two-color image. The quantitative analysis of DCF (green) flu-

Table 1
Quantification of AQs effect over *C. tropicalis* biofilms under darkness and irradiation, expressed in biofilm biomass units (BBU).

		Strain No. 1		Strain No. 2	
		Darkness	Irradiation	Darkness	Irradiation
	BIOFILM	42.59 ± 6.25	44.51 ± 6.70	45.26 ± 3.72	45.10 ± 1.38
AQ1	SubMIC	44.61 ± 7.74	9.57 ± 3.79*,#	46.30 ± 5.02	46.08 ± 3.14
	MIC	41.30 ± 1.98	11.78 ± 3.76*,#	45.14 ± 2.95	25.26 ± 4.58*,#
	SupraMIC	35.18 ± 7.32	7.98 ± 1.35*,#	43.45 ± 3.05	16.46 ± 2.04*,#
AQ2	SubMIC	44.61 ± 7.74	11.42 ± 1.08*,#	45.49 ± 3.85	44.63 ± 1.68
	MIC	41.30 ± 1.98	9.48 ± 2.49*,#	46.57 ± 5.42	23.56 ± 7.79*,#
	SupraMIC	38.45 ± 2.99	7.98 ± 1.35*,#	45.47 ± 2.29	42.17 ± 3.01

* $p < 0.005$ vs untreated biofilm;
\# $p < 0.005$ irradiation vs. darkness.

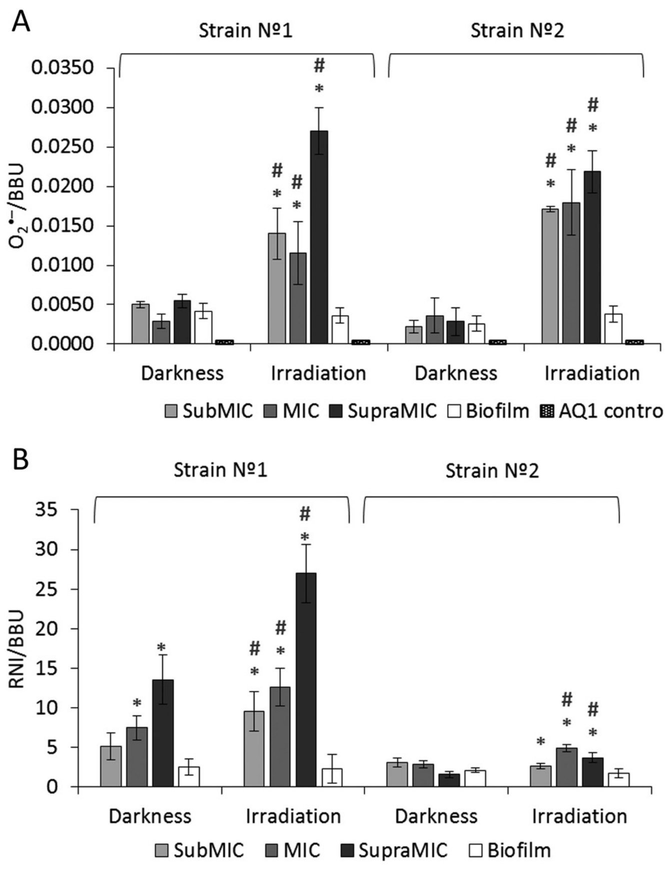

Fig. 3. ROS and RNI generation by AQ1 in *C. tropicalis* biofilms, under darkness and irradiation: (A) ROS/BBU ratio determined by NBT assay, (B) RNI/BBU ratio determined by Griess method. Error bars represent the standard deviations of the means of three independent experiments. * denotes statistical significance at $p < 0.005$ when compared to untreated biofilms. # denotes statistical significance at $p < 0.005$ when darkness and irradiation were compared.

orescence intensities were 1.2 ± 0.2 for AQ1 and 0.4 ± 0.1 for the control.

Regarding the protective antioxidant mechanisms, a stimulation of SOD activity in biofilms was observed and this stimulation was proportionally dependent on AQ1 concentrations in both strains (Fig. 5A). In strain No. 1, SOD stimulation was observed in darkness as well as under irradiation (*$p < 0.005$). Moreover, SOD activity was high in response to the great ROS levels shown previously in Fig 3A, especially at Supra MIC by light action. On the other hand, in strain No. 2, the enzyme activity was only stimulated under irradiation, showing that the clinical strain have better antioxidant response. In addition, FRAP assay was performed in order to assess the biofilm's total antioxidant capacity that includes enzymatic and non-enzymatic defenses. FRAP was only enhanced by light action (Fig. 5B). At Supra MIC, low FRAP levels were observed in both strains, probably due to the high levels of oxidative metabolites under irradiation. Therefore, the total antioxidant capacity of the biofilm may not be enough to counteract the cellular stress, resulting in BBU reduction without its eradication.

Table 2
Fractional inhibitory concentration (FIC) and FIC index (FICI) of AQs combined with AmB against *C. tropicalis* biofilms under irradiation.

Strain No. 2	SMICa (µg/ml)	SMICb (µg/ml)	FIC	FICI
AQ1- AMP-B				
AMP-B (µg/ml)	0.5	0.25	0.5	
AQ1 (µg/m)	1.96	0.49	0.25	0.75
AQ2- AMP-B				
AMP-B (µg/ml)	0.5	0.25	0.5	
AQ2 (µg/ml)	15.6	7.81	0.5	1

SMICa represents the SMIC values of AQ1, AQ2 or AmB alone and SMICb is the SMIC value of AQ1, AQ2 or AmB in the combination.

From the antifungal activity assay, the SMIC 50 values for each AQ and AmB were estimated, and the SMIC 50 values for the combinations of each AQ with AmB were obtained by checkerboard microdilution method with the aim to study the effects of antifungal combinations. FIC and FICI of AQ1 or AQ2 combined with AmB against *C. tropicalis* biofilms under irradiation were calculated (Table 2). The SMIC 50 of AmB was reduced from 0.5 to 0.25 µg/ml when it was combined with either anthraquinone (AQ1 or AQ2) for both strains, whereas the individual SMIC 50 value of AQ1 dropped 4-fold in combination with AmB for *C. tropicalis* biofilms. However, SMIC 50 value of AQ2 dropped 2-fold in combination with AmB for *C. tropicalis* biofilms. Therefore, AmB/AQ1 combination yielded a partial synergy against strain No. 2 biofilms and AmB/AQ2 an addition effect. The same results were obtained for strain No. 1 (data not shown).

Discussion

C. tropicalis is increasingly becoming among the most commonly isolated pathogens causing fungal infections and the capacity to form biofilms is a challenge to clinical treatment (Chandra et al., 2012). Several reports show that *Candida* species biofilms are resistant to most of the commonly used antifungal drugs and various reasons have been proposed to be responsible for the antifungal resistance (Tobudic et al., 2012; Olsen, 2015).

In the present study, antifungal activity of two AQs (1 and 2) against *C. tropicalis* biofilms was investigated. The results demonstrated that AQ1 was more active than AQ2 in planktonic and sessile cells. These natural AQs of *H. pustulata*, having photosensitizing

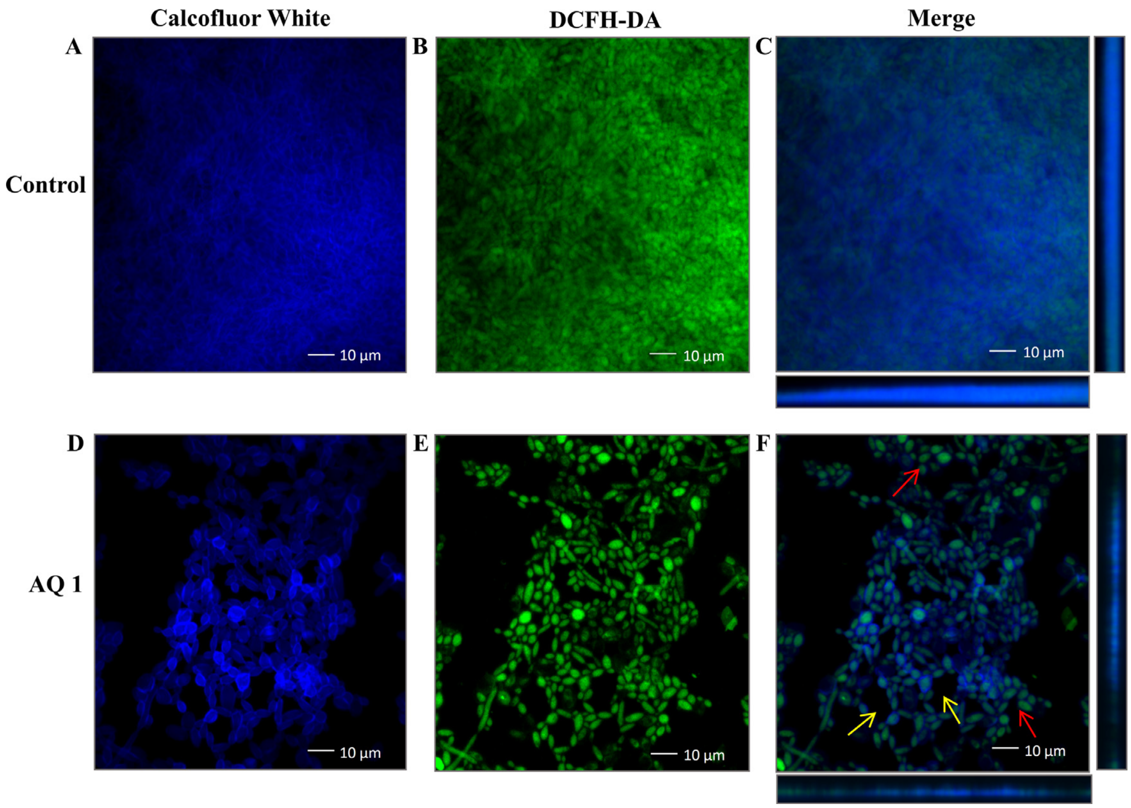

Fig. 4. CSLM images of ROS intracellular *C. tropicalis* biofilms with Rubiadin (AQ1). Blue channel shows Calcofluor in sessile cells walls (A and D), and green channel shows oxidation of the dye DCFH as an indicator of $O_2^{\bullet-}$ production inside biofilms (B and E). Two-color merged image of A and B (C and F). Magnification 60× and scale bar is 10 µm. (For interpretation of the references to colour in this figure legend, the reader is referred to the web version of this article.)

properties, strongly reduced *C. tropicalis* biofilm under light action; thus it could establish their potential use in Antimicrobial Photodynamic Therapy (Pereira Gonzalez and Maich, 2012). As the photosensitizing properties involve ROS generation (Núñez Montoya et al., 2005), this was evaluated as an action mechanism. Cellular redox homeostasis is very important for microbial survival and situations that cause an imbalance between the ROS production and the antioxidant defenses levels can affect the microbial growing and viability (Mishra and Imlay, 2012; Peralta et al., 2015). Although adaptive responses against oxidative stress caused by this ROS overproduction have been extensively studied in planktonic cells, comparatively little is known about the biofilm responses and the basis for this apparent acquired resistance are currently unknown. In biofilms treated with AQ1, ROS production was significantly increased in both strains under irradiation, confirming its photosensitizing properties also in biofilms. Similar patterns of stress metabolites (ROS and RNI) were found in biofilms of both strains, showing an increase of these species directly proportional to AQ1 concentrations. A significant difference between darkness and irradiation conditions was observed, with a lower production of stress metabolites in darkness. In addition, as response to the cellular stress, SOD levels increased significantly, especially at supra-MICs.

Nitrosative stress occurs when the production of NO or other RNI increase in important amounts. NO reacts with oxygen, $O_2^{\bullet-}$ and reducing agents to generate other products resulting in toxic events that if they overwhelm the ability of the cell to remove them, an irreversible damage occurs in several cellular components (Hughes, 2008). In this work we have demonstrated that the clinical strain generated higher levels of NO than the reference strain. This could be related to its low total antioxidant capacity determined by FRAP, since other results in eukaryotic cells have indicated that chemical depletion of antioxidant defences significantly enhanced the cytotoxicity of NO, and high levels of non-enzymatic antioxidant defences are critical for cellular protection against the RNI effects (Ridnour et al., 2004). Therefore, it was necessary to consider several factors to understand the REDOX imbalance in biofilms, to counteract the oxidative stress generated by AQs. In this work, a possible decrease in FRAP at supra MIC was studied as another factor involved in imbalance. We postulated that the antifungal effect of AQ1 could be explained by Redox imbalance that interferes on cellular growth and viability inside of biofilms, enhanced by the action of light.

Another therapeutic strategy that is being developed in the last time with promising results, especially against planktonic strains of *Candida* spp. (Tobudic et al., 2012), is the combination of drugs with different mechanisms of action, since this therapy shows the advantage to inhibit multiple cellular targets. Consequently, combination therapy would be a good strategy against biofilms because it could act on several factors that contribute to biofilm resistance. The results obtained with planktonic forms may not always work in biofilm setup, and therefore the drugs combination must be studied in biofilms. Therefore, with the aim to enhance the antifungal effect of these natural AQs, the interaction of each AQ with AmB against *C. tropicalis* biofilms in vitro was evaluated by using the checkerboard microdilution method. A significant decrease in the SMIC 50 values was obtained for the combinations of each AQ with AmB, demonstrating a synergic effect on the biofilm reduction by light action. The synergistic interaction between AQs and AmB could be explained by the different action mechanisms of each compound: the photosensitizing effect of AQs and the cell membrane disruption in biofilms by AmB that helps the penetration of the AQs.

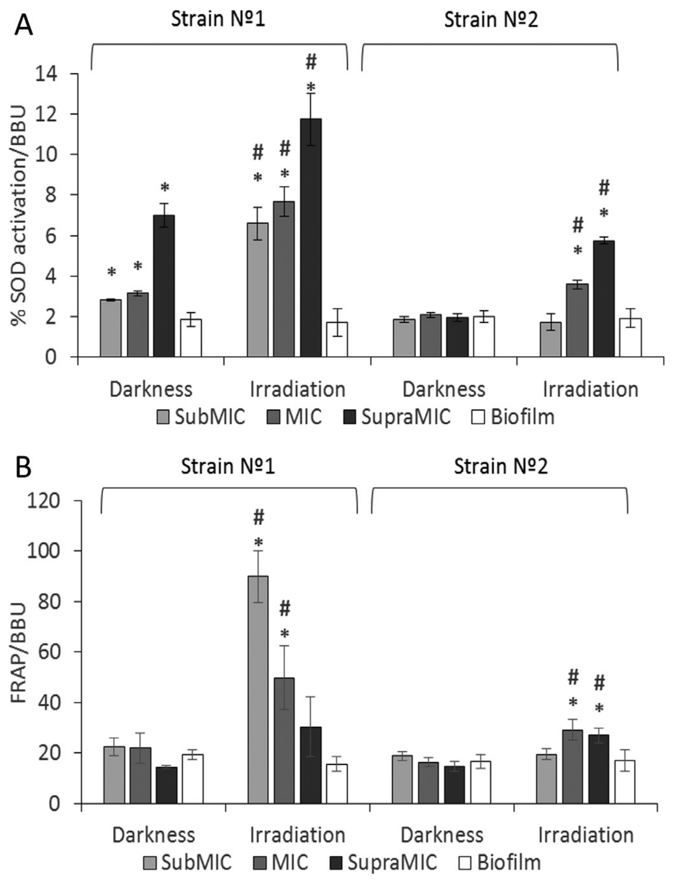

Fig. 5. Antioxidant system activation by AQ1 in *C. tropicalis* biofilms, under darkness and irradiation. (A) SOD activation (%) / BBU. (B) FRAP/ BBU. Error bars represent the standard deviations of the means of three independent experiments. * denote statistical significance at $p < 0.005$ when compared to untreated biofilms. # $p < 0.005$ dark versus light conditions.

The combined use of antifungal drugs with photosensitizing compounds may improve the treatment of infections associated to *C. tropicalis* biofilms, by disrupting biofilms and thus preventing the emergence of resistance. To our knowledge, studies of drug combination in biofilms are scarce, and so far none have evaluated the effect of light in the combination. Thus, the in vitro phenomenon of synergism of AQ1 and AmB against *C. tropicalis* biofilms under irradiation is reported here for the first time. Moreover, the evidence of synergism in this antifungal combination therapy in vitro might be the first step in establishing an appropriate antibiofilm therapy (Sardi et al., 2013). However, much more studies are necessary in order to explore their toxicity to mammalian cells and drug-like properties.

Conclusions

Biofilm infections are particularly difficult to eradicate and the most used available antifungals have a limited activity in them. Therefore, the discovery of novel compounds and innovative strategies to treat fungal biofilms is of great interest. The results presented here show the synergistic activity of AQ1 with an antifungal drug widely used in therapy (AmB) against *C. tropicalis* biofilm. This study shows for the first time that the combination of AQs sensitizes sessile cells of biofilms. Further studies in this direction would give insight into an effective strategy for clinical applicability in prophylaxis and treatment of infections associated to *C. tropicalis* biofilm.

Conflicts of interest

The authors declare that they have no conflict of interest.

Acknowledgments

S. C. Núñez Montoya, J. L. Cabrera, and M. G. Paraje are members of the Research Career of CONICET. J. Marioni and M. A. da Silva are doctoral fellow of CONICET. The authors wish to thank Dra. A. Trenchi, Dra. M. C. Sampedro and Dr. C. Mas for their excellent technical assistance in CSLM. This work was supported by the following grants: SECyT, FONCyT, MinCyT and CONICET. We thank Dr. Paul Hobson, native speaker, for revision of the manuscript.

Supplementary materials

Supplementary material associated with this article can be found, in the online version, at doi:10.1016/j.phymed.2016.07.008.

References

Al-Fattani, M.A., Douglas, L.J., 2006. Biofilm matrix of *Candida albicans* and *Candida tropicalis*: chemical composition and role in drug resistance. J. Med. Microbiol. 55, 999–1008.
Angel Villegas, N., Baronetti, J.L., Albesa, I., Etcheverría, A., Becerra, M.C., Padola, N.L., Paraje, M.G., 2015. Effect of antibiotics on cellular stress generated in Shiga toxin-producing *Escherichia coli* O157: H7 and non-O157 biofilms. Toxicol. in Vitro 29, 1692–1700.
Arce Miranda, J.E., Sotomayor, C.E., Albesa, I., Paraje, M.G., 2011. Oxidative and nitrosative stress in *Staphylococcus aureus* biofilm. FEMS Microbiol. Lett. 1, 23–29.
Baronetti, J.L., Angel Villegas, N., Paraje, M.G., Albesa, I., 2011. Nitric oxide-mediated apoptosis in rat macrophages subjected to Shiga toxin 2 from *Escherichia coli*. Microbiol. Immunol. 55, 231–238.
Berg, D., Youdim, M.B.H., Riederer, P., 2004. Redox imbalance. Cell Tissue Res. 318, 201–213.
Bergamo, V.Z., Balbueno, E.A., Hatwig, C., Pippi, B., Lana, D.F., Donato, R.K., Schrekker, H.S., Fuentefria, A.M., 2015. 1-n-Hexadecyl-3-methylimidazolium methanesulfonate and chloride salts with effective activities against *Candida tropicalis* biofilms. Lett. Appl. Microbiol. 61, 504–510.
Chandra, J., Mukherjee, P.K., Hoyer, L.L., Ghannoum, M.A., 2012. *Candida* biofilms associated with CVC and medical devices. Mycoses. 55, 46–57.
Clinical and Laboratory Standards Institute (CLSI), 2008. Reference Method for Broth Dilution Antifungal Susceptibility Testing of Yeasts, Approved Standard—third ed Clinical and Laboratory Standards Institute, USA CLSI Document M27-A3. 950 West Valley Road, Suite 2500, Wayne, Pennsylvania 19087.
Comini, L.R., Fernandez, I.M., Rumie Vittar, N.B., Núñez Montoya, S.C., Cabrera, J.L., Rivarola, V.A., 2011. Photodynamic activity of anthraquinones isolated from *Heterophyllaea pustulata* Hook f. (Rubiaceae) on MCF-7c3 breast cancer cells. Phytomedicine. 18, 1093–1095.
Dastgheyb, S.S., Eckmann, D.M., Composto, R.J., Hickok, N.J., 2013. Photo-activated porphyrin in combination with antibiotics: therapies against Staphylococci. J. Photochem. Photobiol. B. 129, 27–35.
Delattin, N., Cammue, B.P., Thevissen, K., 2014. Reactive oxygen species inducing antifungal agents and their activity against fungal biofilms. Future Med. Chem. 6, 77–90.
Hughes, M.N., 2008. Chapter One- Chemistry of nitric oxide and related species. Methods Enzimol. 436, 3–19.
Marioni, J., Arce Miranda, J.E., Cabrera, J.L., Paraje, M.G., Núñez Montoya, S.C., 2016. Reduction of *Candida tropicalis* biofilm by photoactivation of a *Heterophyllaea pustulata* extract. Pharm. Biol. http://dx.doi.org/10.1080/13880209.1183683.
Mah, T., 2012. Biofilm-specific antibiotic resistance. Future Microbiol. 7, 1061–1072.
Messier, C., Epifano, F., Genovese, S., Grenier, D., 2011. Inhibition of *Candida albicans* biofilm formation and yeast-hyphal transition by 4-hydroxycordoin. Phytomedicine. 18, 380–383.
Mishra, S., Imlay, J., 2012. Why do bacteria use so many enzymes to scavenge hydrogen peroxide? Arch. Biochem. Biophys. 525, 145–160.
Núñez Montoya, S.C., Agnese, A.M., Perez, C., Tiraboschi, I.N., Cabrera, J.L., 2003. Pharmacological and toxicological activity of *Heterophyllaea pustulata* anthraquinone extracts. Phytomedicine. 10, 569–574.
Núñez Montoya, S.C., Comini, L.R., Sarmiento, M., Becerra, C., Albesa, I., Argüello, G.A., Cabrera, J.L., 2005. Natural anthraquinones probed as Type I and Type II photosensitizers: singlet oxygen and superoxide anion production. J. Photochem. Photobiol. B. 78, 77–83.
O'Toole, G.A., Kolter, R., 1998. Initiation of biofilm formation in *Pseudomonas fluorescens* WCS365 proceeds via multiple, convergent signaling pathways: a genetic analysis. Mol. Microbiol. 28, 449–461.
Olsen, I., 2015. Biofilm-specific antibiotic tolerance and resistance. Eur. J. Clin. Microbiol. Infect. Dis. 34, 877–886.

Peralta, M.A., da Silva, M.A., Ortega, M.G., Cabrera, J.L., Paraje, M.G., 2015. Antifungal activity of a prenylated flavonoid from *Dalea elegans* against *Candida albicans* biofilms. Phytomedicine. 22, 975–980.

Pereira Gonzalez, F., Maich, T., 2012. Photodynamic inactivation for controlling *Candida albicans* infections. Fungal Biol. 116, 1–10.

Pfaller, M.A., 2012. Antifungal drug resistance: mechanisms, epidemiology, and consequences for treatment. Am. J. Med. 125 S3–S13.

Pierce, C.G., Uppuluri, P., Tristan, A.R., Wormley Jr, F.L., Mowat, E., Ramage, G., Lopez-Ribot, J.L., 2008. A simple and reproducible 96-well plate-based method for the formation of fungal biofilms and its application to antifungal susceptibility testing. Nat. Protoc. 3, 1494–1500.

Ramage, G., Rajendran, R., Sherry, L., Williams, C., 2012. Fungal biofilm resistance. Int. J. Microbiol. 2012, 528521.

Raman, N., Lee, M.R., Lynn, D.M., Palecek, S.P., 2015. Antifungal activity of 14-Helical β-peptides against planktonic cells and biofilms of *Candida* species. Pharmaceuticals. 8, 483–503.

Ridnour, L.A., Thomas, D.D., Mancardi, D., Espey, M.G., Miranda, K.M., Paolocci, N., Feelisch, M., Fukuto, J., Wink, D.A., 2004. The chemistry of nitrosative stress induced by nitric oxide and reactive nitrogen oxide species. Putting perspective on stressful biological situations. Biol. Chem. Jan. 385, 1–10.

Sardi, J.C.O., Scorzoni, L., Bernardi, T., Fusco-Almeida, A.M., Mendes Giannini, M.J.S., 2013. *Candida* species: current epidemiology, pathogenicity, biofilm formation, natural antifungal products and new therapeutic options. J. Med. Microbiol. 62, 10–24.

Silva-Dias, A., Miranda, I.M., Branco, J., Monteiro-Soares, M., Pina-Vaz, C., Rodrigues, A.G., 2015. Adhesion, biofilm formation, cell surface hydrophobicity, and antifungal planktonic susceptibility: relationship among *Candida* spp. Front. Microbiol. 6, 205.

Tobudic, S., Kratzer, C., Lassnigg, A., Presterl, E., 2012. Antifungal susceptibility of *Candida albicans* in biofilms. Mycoses 55, 199–204.

Uppuluri, P., Pierce, C.G., Lopez-Ribot, J.L., 2009. *Candida albicans* biofilm formation and its clinical consequences. Future Microbiol. 4, 1235–1237.

Vandenbosch, D., Braeckmans, K., Nelis, H.J., Coenye, T., 2010. Fungicidal activity of miconazole against *Candida* spp. biofilms. J. Antimicrob. Chemother. 65, 694–700.
